# Short-chain polyphosphates induce tau fibrillation and neurotoxicity in human iPSC-derived retinal neurons

**DOI:** 10.1038/s41419-025-07662-5

**Published:** 2025-05-09

**Authors:** Lorenzo Barolo, Lorenza Mautone, Ylenia Gigante, Silvia Ghirga, Francesco Mura, Maria Vittoria Farina, Stefano Tacconi, Luciana Dini, Giancarlo Ruocco, Alberto Boffi, Edoardo Milanetti, Silvia Di Angelantonio, Paola Baiocco

**Affiliations:** 1 Department of Biochemical Sciences “Alessandro Rossi Fanelli”, Sapienza University of Rome, Rome, Italy; 2 D-Tails srl BC, Rome, Italy; 3 Center for Life Nano- & Neuro-Science@Sapienza, Istituto Italiano di Tecnologia, Rome, Italy; 4 Department of Physiology and Pharmacology, Sapienza University of Rome, Rome, Italy; 5 Research Center on Nanotechnologies Applied to Engineering of Sapienza (CNIS), Sapienza University of Rome, Rome, Italy; 6 Department of Biology and Biotechnologies, Sapienza University of Rome, Rome, Italy; 7 Department of Physics, Sapienza University of Rome, Rome, Italy

**Keywords:** Stem cells in the nervous system, Proteins

## Abstract

The onset of Alzheimer’s Disease and Frontotemporal Dementia is closely associated with the aggregation of tau, a multifunctional protein essential for neuronal stability and function. Given the role of tau aggregation in neurodegeneration, understanding the mechanisms behind its fibril formation is crucial for developing therapeutic interventions to halt or reverse disease progression. However, the structural complexity and diverse aggregation pathways of tau present significant challenges, requiring comprehensive experimental studies. In this research, we demonstrate that short-chain polyphosphates, specifically sodium tripolyphosphate (NaTPP), effectively induce tau fibril formation in vitro using the microtubule-binding domain fragment (K18). NaTPP-induced fibrils display unique structural characteristics and aggregation kinetics compared to those induced by heparin, indicating distinct pathogenic pathways. Through molecular dynamics simulations, we show that NaTPP promotes aggregation by exposing key residues necessary for fibril formation, which remain concealed under non-aggregating conditions. This interaction drives tau into an aggregation-prone state, revealing a novel mechanism. Furthermore, our study indicates that human pluripotent stem cell-derived retinal neurons internalize NaTPP-induced fibrils within 24 h, pointing to a potential pathway for tau spread in neurodegeneration. To explore the translational implications of NaTPP-induced fibrils, we assessed their long-term effects on cellular viability, tubulin integrity, and stress responses in retinal neuron cultures. Compared to heparin, NaTPP promoted fewer but longer fibrils with initially low cytotoxicity but induced a stress response marked by increased endogenous tau and p62/SQSTM1 expression. Prolonged exposure to NaTPP-induced oligomers significantly increased cytotoxicity, leading to tubulin fragmentation, altered caspase activity, and elevated levels of phosphorylated pathological tau. These findings align with a neurodegenerative phenotype, highlighting the relevance of polyphosphates in tau pathology. Overall, this research enhances our understanding of the role of polyphosphate in tau aggregation, linking it to key cellular pathways in neurodegeneration.

## Introduction

Tau is a microtubule-associated protein that plays a crucial role in maintaining axonal structural integrity in neurons [1, 2]. Its dysregulation leads to neurofibrillary tangles (NFTs), protein aggregates characteristic of neurodegenerative diseases such as Alzheimer’s disease (AD) and frontotemporal dementia (FTD) [3–6]. These pathologies are primarily driven by tau oligomers, which accumulate intracellularly during fibril formation and spread between neurons, contributing to disease progression [7–10].

Recent studies have demonstrated that tau oligomers generated in vitro can be internalized by neurons derived from induced pluripotent stem cells (iPSCs) and murine models [11–13]. Once internalized, these oligomers act as templates, seeding the aggregation of endogenous tau and amplifying pathological tau deposition [14–17]. To model these aggregation dynamics, the microtubule-binding repeat region (MTBR), or K18 domain [18–20] is typically incubated with polyanionic compounds like polyunsaturated fatty acids, RNA, and polyglutamate [3, 21–24]. Heparin is frequently used for this purpose, but its use presents significant limitations, as it does not participate in tau fibrillation in vivo, and its polymorphic nature complicates the study of tau behavior under physiological conditions [21, 25].

Polyphosphates (polyPs), naturally present in the brain, are involved in cellular energy homeostasis, inflammation, and cell signaling. Notably, polyPs can compete with tubulin for tau binding, thereby promoting its fibrillation [26–34]. Here, we present novel evidence demonstrating that sodium tripolyphosphate (NaTPP), a well-defined polyP analog with a net charge of −5, can drive the formation of mature K18 tau fibrils. Unlike polydisperse polyP mixtures, NaTPP defined structure allows precise analysis of its role in tau aggregation. Given its structural similarity to ATP, which has been shown to catalyze tau fibrillation [35], NaTPP serves as a valuable tool for studying the molecular mechanisms of tauopathies.

Our findings reveal that NaTPP-induced fibrils differ significantly from those generated by heparin in aggregation kinetics, filament length, and structure. Molecular dynamics simulations suggest that NaTPP facilitates tau aggregation by exposing key fibril-forming residues, particularly the GGG motif, which remains buried in non-aggregating conditions. In human iPSC-derived retinal neurons, NaTPP-induced fibrils are internalized within 24 h, potentially contributing to tau propagation in neurodegeneration.

In the long-term, NaTPP-induced fibrils lead to heightened cytotoxicity, tubulin fragmentation, caspase activation, and pathological tau phosphorylation, hallmarks of neurodegeneration. Based on these findings, short polyP may facilitate K18-fibril formation and neuronal uptake, potentially promoting tau-related neurodegeneration.

## Materials and methods

### Tau protein construct design, expression and purification

**Fig. 1:**
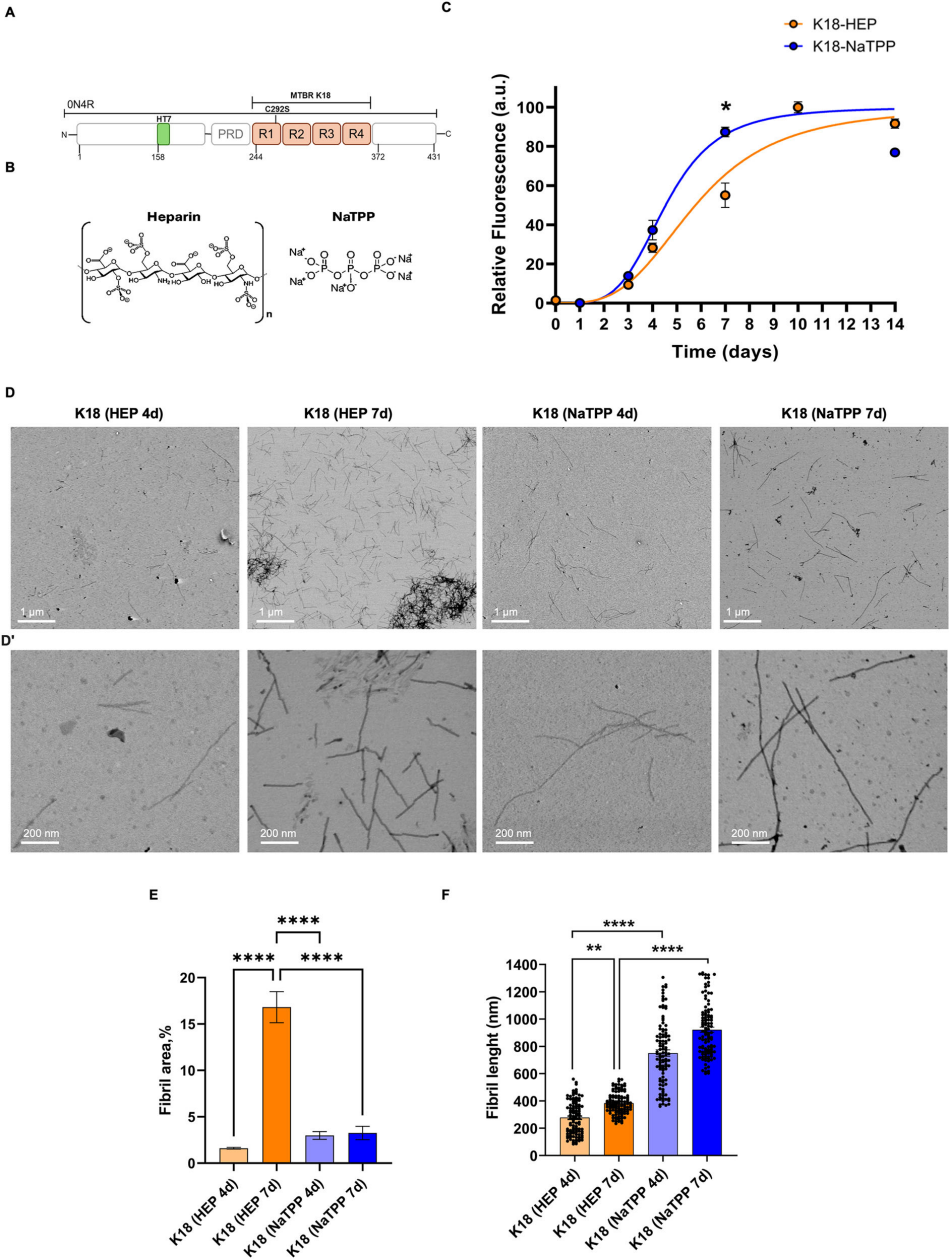
Kinetics of aggregation and structural characterization over time of K18 domain with heparin and NaTPP cofactors. **A** Schematic view of the full-length form of protein tau (0N4R). Recombinant K18 domain from 244-372 and the mutation C249S are shown. HT7 recognition site is also indicated in green; **B** Chemical structures of anionic compounds used in the same ratio 1:1 = protein:cofactor; **C** Representative plot showing the fibril formation using BT1 fluorescence as a function of time in the presence of non-fibrillated K18, 100 µM, and fibrillated K18-Hep, 100 µM (orange curve) and K18-NaTPP 100 µM (blue curve) at 37 °C at different time points. All spectra were obtained using a λex = 530 nm, λem = 565 nm. All data are mean ± SEM, n = 3, two-way ANOVA and Sidak’s multiple comparisons post hoc test *p < 0.05; Representative STEM images of negative stained K18 fibrils, at lower (**D**) and higher (**D**’) magnification (scale bars 1 μm and 200 nm, respectively). The fibrils were formed with heparin after 4 days (i) and 7 days (ii), and with NaTPP after 4 days (iii) and 7 days (iv); **E** measurement of population distribution of fibrils in all four samples calculated from STEM images (All data are mean ± SEM, n=FOV = 3, one-way ANOVA and Tukey’s multiple comparisons post hoc test ****p < 0.0001); **F** measurement of average length of K18 fibrils in all four samples calculated from STEM images (All data are mean ± SEM, *n* = 50, Kruskal Wallis and Dunn’s multiple comparisons post hoc test **p < 0.01, ****p < 0.0001).

This study used the K18 domain referred to as the MTBR domain of tau isoform 0N4R (residues 244 to 372) (Fig. 1A) containing a point mutation C291S [36–38]. Production and purification of the wild type and the K298A, the Q307A and the K298A-Q307A mutants are described in Supplementary Information (SI) [39, 40]. The conformational assembly of K18 was analyzed by size exclusion chromatography using a HiLoad™ 26/600 Superdex 75 (Cytiva).

### Formation and analysis of K18 fibrils

K18 and mutants (100 µM) in PBS were reduced using 1 mM TCEP 10 min at 55 °C. Sodium heparin or NaTPP was added in a 1:1 ratio to initiate tau protein aggregation in vitro. The samples (K18-Hep and K18-NaTPP) were kept at 37 °C under rotation at 150 rpm for several time points. The fibrillation kinetics was analyzed using the BODIPY-based probe BT1, loaded into humanized ferritin nanocages due to the limited specificity of thioflavin T (ThT) towards K18 fibrils [41–43]. All the measurements were made by using the RF-6000 fluorimeter (Shimadzu RF-6000). Further details and scanning transmission electron microscopy (STEM) imaging are given in SI.

### Computational methods

For each of the molecular systems considered in this work, we used Gromacs 2020 [44] and built the system topology using the CHARMM-36 force field [45] using the predicted model obtained by the AlphaFold2 algorithm [46] as the initial protein structure for the simulation. All the details of the performed simulations are available in the SI.

### Retinal neuron differentiation from human iPSC and treatment

Human iPSCs (SIGi001-A) were differentiated into retinal neurons using a modified multi-step protocol [47–49] detailed in SI. DIV 30 retinal neurons were treated with 5 μM of fibrillated K18 samples for 24 h at 37 °C and 5% CO_2_. The effects of this treatment were evaluated 24 h or two weeks, as detailed in Supplementary Information.

### Cytotoxicity analysis

A live-dead assay was conducted with Fluoresceine and Propidium Iodide. Percentages of live (FDA positive cells) and dead cells (PI positive cells) were calculated as (Live/Total Cells) * 100 and (Dead/Total Cells) * 100, after 24 h and 2 weeks.

### Immunostaining, confocal imaging, and analysis

Retinal cells were fixed with 4% PFA and incubated overnight with antibodies: TUJ1, pTAU Ser202/Ser205, HT7, p62/SQSTM1, MAP2, and Cleaved caspase 3, followed by AlexaFluor secondary antibodies and Hoechst. Confocal images (2048 × 2048) were acquired using a spinning disk X-Light V3 (CrestOptics) confocal microscope. Cytoskeleton integrity was measured as the integrated density of TUJ1 and MAP2, with segment analysis for TUJ1. Total tau quantification was performed as the integrated density of HT7. Phospho-Tau Ser202/Ser 205 and P62/SQSTM1 were analyzed as puncta, and colocalization with rhodamine-K18 was assessed using Mander’s overlap coefficient. Cleaved-caspase 3 positive nuclei were manually counted and calculated as (cleaved-caspase 3 positive cells/total cells) * 100. Nuclear morphology was analyzed by assessing nuclear size and density [49, 50].

### Statistics

The analysis was conducted on at least three biological replicates per condition. Data were reported as mean ± SEM and plotted using GraphPad Prism 8.0. Statistical analysis involved parametric or non-parametric ANOVA, with treatment as the independent variable. Significant differences were further analyzed using Dunnett, Sidak, or Tukey multiple comparison tests. Significance levels were *p ≤ 0.05, **p ≤ 0.01, ***p ≤ 0.001, ****p ≤ 0.0001.

## Results

### Heparin- and NaTPP-induced K18 fibrils show different fibrillation kinetics and morphology

To evaluate the effects of the two different aggregation agents, heparin and NaTPP (Fig. 1B), the fibrillated samples were analyzed using a recently developed probe with high affinity for tau-based β-sheet structures, BT1-loaded ferritin (HumAfFt-BT1) [41, 42]. Different aliquots of fibrils were taken from each sample at various time points and analyzed by HumAfFt-BT1 fluorescence assay (Fig. 1C). In our experiment, the initial lag phase for both K18-Hep and K18-NaTPP corresponded to 3 days of incubation at 37 °C [28]. Over this period, the samples showed a negligible increase in fluorescence due to the formation of numerous nuclei (Fig. 1C). However, after 4 days, both samples reached the growth phase corresponding to the elongation of the nuclei into oligomers and protofibrils. The fluorescence intensity increased similarly for both K18-Hep and K18-NaTPP. The elongation of the fibrils continued up to the seventh day, in which a significant difference in fluorescence between the samples was observed (Fig. 1C; K18-Hep-7d VS K18-NaTPP-7d *p* = 0.0182). K18-NaTPP showed a higher fluorescence intensity, almost double the one of K18-Hep. After 10 days, the growing phase ended, and the fluorescence intensity remained constant, showing a slight decrease after 14 days, probably caused by the formation of very insoluble aggregates (Fig. 1C). The major differences in fibrillation kinetics were observed during the growth phase, between 4 and 7 days. Therefore, to explore the effects of the NaTPP on the formation and morphology of K18 fibrils, these two timepoints were further investigated by scanning transmission electron microscopy (STEM). K18 exposed to heparin (1:1 in molar concentrations) showed great difference after 4 and 7 days (Fig. 1D–F). Population distribution of the K18-Hep sample after 4 days at 37 °C resulted in a scarce amount of fibrils (1.6 ± 0.13%), while after 7 days the population of fibrils was richer (19.3 ± 4.25%) (Fig. 1E; K18-Hep-4d VS K18-Hep-7d *p* < 0.0001). The differences between these two samples concerned also the length of the fibrils. In fact, after 4 days of exposure to heparin, K18 presented higher fibril variability, whereas the protein showed multiple aggregates with a more consistent and final fibril length after 7 days (Fig. 1F; K18-Hep-4d VS K18-Hep-7d *p* = 0.0093). This behavior was similar for K18-NaTPP samples. On average, these fibrils were longer than K18-Hep fibrils (K18-NaTPP > 800 nm, K18-Hep < 400 nm, Fig. 1F). However, after 4 days at 37 °C, the fibrils still showed oscillations in dimension, whilst after 7 days, the length variability was lower, although still present (Fig. 1F; K18-NaTPP-4d VS K18-NaTPP-7d). The population distribution of K18-NaTPP fibrils after 4 days (2.94 ± 0.72%) was similar to the same sample after 7 days (3.28 ± 1.25%) (Fig. 1E). No fibrils were observed in control experiments with NaP (Supplementary Fig. S2). These data indicate that K18 exposed to NaTPP produces longer fibrils than heparin, showing fibril maturity after 7 days.

### Molecular dynamics simulations reveal NaTPP-induced aggregation mechanisms of K18 tau protein

With the aim of investigating the equilibrium conformational exploration of K18, we performed a one-microsecond molecular dynamics simulation of K18 in the presence of NaTPP. As a control, we also performed two additional one-microsecond molecular dynamics simulations, considering K18 protein alone in the water and K18 in the presence of sodium monophosphate (Na_2_HPO_4_)(NaP).

**Fig. 2:**
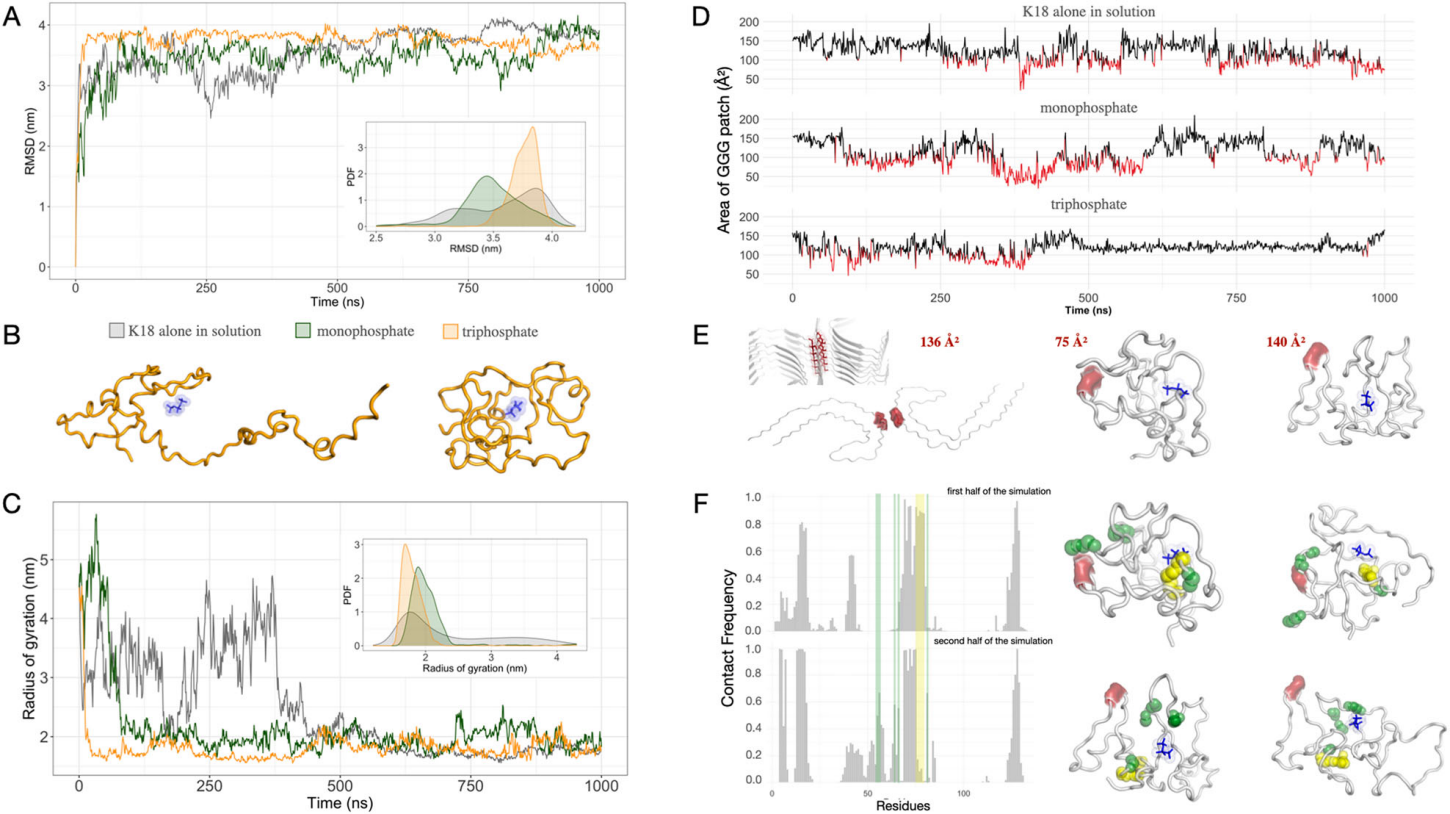
Molecular dynamics simulations analysis. **A** For each of the three simulated systems (K18 in water in gray, K18 with NaP, in green, and K18 with NaTPP, in orange), the RMSD values as a function of time are reported. Additionally, the probability density functions (PDF) of the RMSD values related to the three systems are shown in the inset. **B** Two cartoon representations of two snapshots from the K18 with NaTPP simulation are shown, one with high (less compact form) and one with low radius of gyration (more compact form). **C** Radius of gyration values as a function of time with the corresponding probability density functions in the inset. **D** For each simulated system, we report the surface area of the molecular surface patch formed by the three consecutive glycine residues directly involved in the fibril interface, whose structure is experimentally known. The surface area value, calculated by summing the contributions of the selected residues’ areas, is shown for each frame of the three molecular dynamics simulations. In black are the surface area values greater than 100 Å² (more exposed residues) and in red are the surface area values less than 100 Å² (less exposed residues). **E** On the left is a cartoon representation of the TAU fibril (PDB code 5O3L), with a focus on a single layer of the fibril itself, where the solvent-accessible area of the two sets of three interacting glycines is shown in red. On the right are two cartoon representations of different simulation snapshots: one where the area of the three glycines is below the threshold, and one where the area is above the threshold, showing greater exposure. **F** On the left are the relative contact frequencies between each residue of the K18 protein and the monophosphate molecule. The top barplot corresponds to the first half of the simulation, and the bottom barplot corresponds to the second half of the simulation. On the right are snapshots of the K18 with NaTPP simulation: two snapshots from the first half of the simulation (where the residues shown in green interact with the three glycines involved in the fibril interface) and two snapshots from the second half of the simulation (where the same residues shown in green interact with the ligand). In yellow are the residues interacting with the ligand only in the first part of the simulation.

A set of preliminary analyses (Root Mean Square Deviation (RMSD), radius of gyration, and contact analysis, Fig. 2A–C) for all three simulated systems highlighted that the K18 protein adopts a more compact conformation, regardless of the presence of ligands (see Supplementary Material for more details).

To investigate the dynamic-structural stability of the solvent-exposed regions involved in molecular binding, in this specific case, we leverage the experimental knowledge of the system [6]. Therefore, we selected the three interacting glycines (GGG patch: residues 333, 334, and 335) from PDB 5O3L, which are central residues of the interface. A cartoon representation of this structure is shown in Fig. 2E (left), where the three glycines in question are highlighted in red.

We calculated the area of molecular iso-electron density surfaces formed by the three glycines during the molecular dynamics simulation for the three molecular systems separately, with the idea that the larger the area of the considered surface portion, the greater the possibility of interaction.

For the two systems that did not form aggregates (Fig. 2D, free K18, top trace, and K18 with NaP, middle trace), the solvent-exposed area of residues involved in the fibril interface fluctuated significantly throughout the simulation, transitioning from high exposure values (in black), potentially favorable to binding, to low solvent exposure values (in red), indicative of conformations that theoretically hinder binding.

Interestingly, when K18 interacted with NaTPP, after approximately 500 ns, it began to explore conformations that did not present a marked structural stability compared to the first part of the simulation (see RMSD and radius of gyration analysis) but maintained a highly stable area value in the interface region. The evidence of this signal in Fig. 2D (bottom trace) is of great interest, with an average area value of 121 Å², with a standard deviation of 11 Å². In contrast, during the first half of the simulation, the same system exhibited a more pronounced variability in surface area, with an average of 113 Å² and a standard deviation of 24 Å². A structural visualization of the surface areas related to the three glycines is shown in Fig. 2E, where we show a snapshot of the trajectory in which the three glycines were partially buried (Fig. 2E, middle) and one, belonging to the second half of the simulation of K18 in the presence of NaTPP, in which the three glycines are markedly exposed (Fig. 2E, right).

The stabilization of the solvent-exposed area of residues involved in the fibril interface suggests the importance of stabilizing not only the entire protein structure (probably a necessary condition) but also the binding region (a necessary and sufficient condition).

Additionally, based on the ligand-protein distance, we defined the residues in contact with the NaTPP at each frame of the simulation to calculate the relative contact frequency of each residue separately for the first part and the second part of the molecular dynamics simulation (Fig. 2F, left).

In general, the contacts of the K18 residues interacting with NaTPP are largely conserved. However, in Fig. 2F, right, we highlighted in green the residues that have significantly increased the frequency of contact with the ligand in the second half of the simulation, compared to the first part of the trajectory (297I, 298K, 307Q, 308I, and 323G). On the contrary, in yellow, we reported the residues with many contacts in the first part and a loss of contacts in the second half of the trajectory.

The initial residues in the N-terminus of K18 also became consistently interacting in the second half of the simulation, indicating that the new rearrangement of the protein configuration could also be induced by a new interaction between the ligand and the N-terminal of K18.

During the first half of the simulation, when the area of the GGG patch involved in the fibril interface was variable and generally less exposed, this region frequently interacted with two sets of residues, one centered around 297I and 298K residues, and the other centered around 307Q and 308I residues. At the end of the first half of the simulation, a significant conformational change occurred in K18, as shown in the previous analysis.

In the second half of the simulation, the pairs of residues 297I-298K and 307Q-308I, which formed the basis of a loop created in K18, came closer together and interacted with the ligand (thus moving away from the three glycines belonging to the fibril interface). During the first part of the simulation, these residues were not at the base of the loop and were less constrained. On the contrary, the new configuration restricted the length of this region free to move, making it insufficient to prevent the three glycines from interacting with the solvent. In Fig. 2F, snapshots of the simulation are shown, depicting the contact between residues 297I, 298 K, 307Q, and 308I (in green) and the three glycines (in red) during the first phase and their involvement in binding with the NaTPP during the second half of the simulation. This conformation remained locally stable and ensured the constant exposure of the GGG patch, as evident from the molecular surface area plot. The role of these residues was investigated by alanine mutation of residues K298 and Q307, which might be responsible for the electrostatic interaction with NaTPP (see Supplementary Fig. S3A). The molecular dynamics simulation data obtained for the double mutant shows a lower solvent exposure of the GGG patch, which is experimentally involved in the tau fibril interface, compared to the wild-type form, as can also be observed from the cartoon representation of two randomly selected structures considering the equilibrium phases of the two trajectories (see Supplementary Fig. S4). These observations are consistent with the resulting proteins obtained by site-specific mutagenesis of K298A and Q307A. These single mutants were produced, expressed and purified (Supplementary Fig. S3B). Their ability to form fibrils was then assessed by fluorescence with BT1/ferritin system after 7 days of incubation at 37 °C in the presence of NaTPP, similar to the wild type. The results, presented in the bar graph in Supplementary Fig. S5A, show that the two single mutants have a reduced ability to form fibrils compared to the wild type. Although both retain the ability to aggregate into fibrillar structures, STEM imaging of K298A and Q307A revealed significantly altered morphologies. The K298A mutant forms fibrils of limited length, whereas Q307A produces more entangled and less elongated fibrillar aggregates (Supplementary Fig. S5B–D).

### Short-term cellular responses to K18 fibrils: increased endogenous tau and autophagic activity

**Fig. 3:**
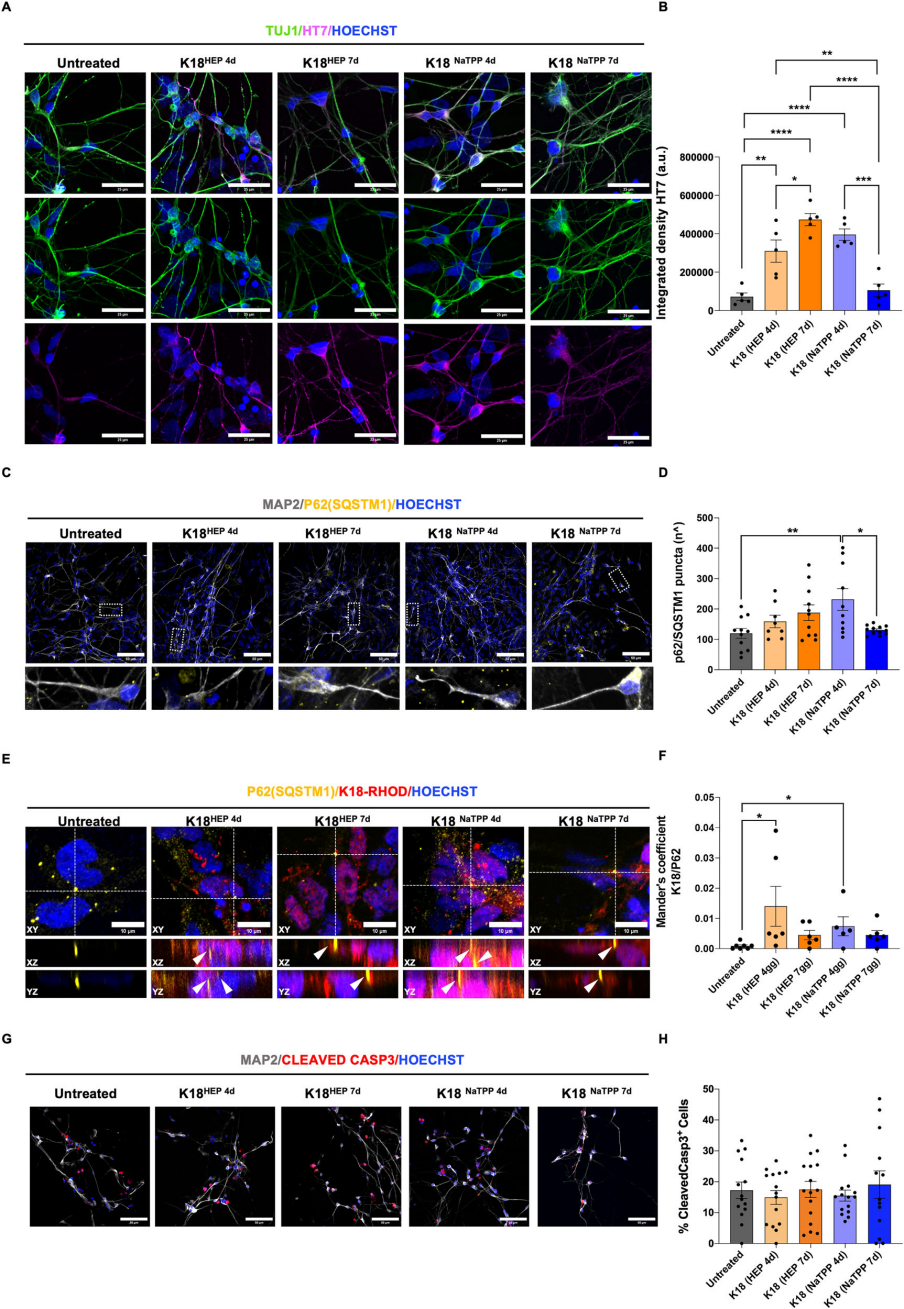
Short-term effects of tau fibrils on iPSC-derived retinal neurons. **A** Confocal immunostaining for total tau HT7 (magenta) and neurite marker β-III-tubulin TUJ1 (green). Nuclei were stained with HOECHST (blue). Scale bar 25 μm; **B** Bar graph represents the integrated density of total tau in retinal neurons 24 hours after K18-tau seeding (n=FOV = 15, one way ANOVA and Tukey’s multiple comparisons post hoc test: K18-Hep-4d VS Untreated *p* = 0.0015, K18-Hep-7d VS Untreated *p* < 0.0001, K18-NaTPP-4d VS Untreated *p* < 0.0001, K18-Hep-4d VS K18-Hep-7d *p* = 0.0357, K18-NaTPP-4d VS K18-NaTPP-7d *p* = 0.0002, K18-Hep-4d VS k18-NaTPP-7d *p* = 0.0065, K18-Hep-7d VS K18-NaTPP-7d *p* < 0.0001; **C** Representative confocal images of retinal neurons immunostained with MAP2 (gray), p62/SQSTM1 (yellow) and HOECHST to stain nuclei (blue). Scale bar 50 µm. Zoomed images show p62 puncta within MAP2 positive neurite; **D** Quantification of p62/SQSTM1 puncta. (n=FOV = 15, one-way ANOVA and Tukey’s multiple comparisons post hoc test: K18-NaTPP-4d VS Untreated *p* = 0.0074, K18-NaTPP-4d VS K18-NaTPP-7d *p* = 0.0162); **E** K18-seed was conjugated with rhodamine (red) and p62/SQSTM1 puncta (yellow) that co-localize with K18 fibrils are highlighted with arrow; Scale bar 10 µm; **F** Bar charts showing the Mander’s colocalization coefficient of k18-fibrils and p62/SQSTM1 in untreated and treated retinal neurons. (n=FOV = 6, Kruskal Wallis and Dunn’s multiple comparisons post hoc test: K18-Hep-4d VS Untreated *p* = 0.0177, K18-NaTPP-4d VS Untreated *p* = 0.0493); **G** Representative confocal images of iPSC-derived retinal neurons stained with MAP2 (gray), CLEAVED-CASPASE 3 (red) and HOECHST (blue). Scale bar 50 µm; **H** Bar graph represents the percentage of cleaved-caspase 3 positive nuclei normalized to the total number of nuclei. (n=FOV = 15, Kruskal–Wallis test, and Dunn’s multiple comparison post hoc test).

To study the impact of K18 fibrils on retinal neurons, we treated DIV 30 human iPSC-derived retinal cultures with 5 µM of K18 fibrils for 24 h after confirming K18 fibril internalization and toxicity (Supplementary Fig. S6, Scheme S6D). Increased endogenous tau protein expression was observed with K18-Hep-4d, K18-Hep-7d, and K18-NaTPP-4d treatments but not with K18-NaTPP-7d. This was verified using an anti-total tau (HT7) antibody that recognizes the “PPGQK” epitope of human tau, absent in K18 (Fig. 1A), thus detecting only endogenous tau (Fig. 3A, B). Interestingly, K18-NaTPP-4d treatment led to the highest expression of endogenous tau protein compared to other samples (Fig. 3B). This increase in tau was not associated with changes in β-III Tubulin (TUJ1) (Supplementary Fig. S7), supporting the hypothesis that exogenous fibrils trigger a stress response, leading to cellular upregulation of endogenous tau [15, 51]. We also observed an increased p62/SQSTM1 puncta following K18 fibril treatment (Fig. 3C, D). Notably, p62 puncta were significantly more abundant in cultures treated with K18-NaTPP-4d than those treated with K18-NaTPP-7d or K18-Hep-4d/7d (Fig. 3D). Additionally, p62 puncta colocalized with exogenous K18 fibrils, with the strongest colocalization observed in K18-Hep-4d and K18-NaTPP-4d treatments (Fig. 3E, F). These results indicate an increase in autophagic flux after 24 h, aligning with p62 neuroprotective role in reducing oligomeric tau. However, we did not observe Cleaved-caspase3 activation, indicating that short-term K18 exposure does not induce apoptosis (Fig. 3G, H).

### Long-term neurodegenerative effects of K18 fibrils: cytoskeleton alteration and apoptotic pathway activation

**Fig. 4:**
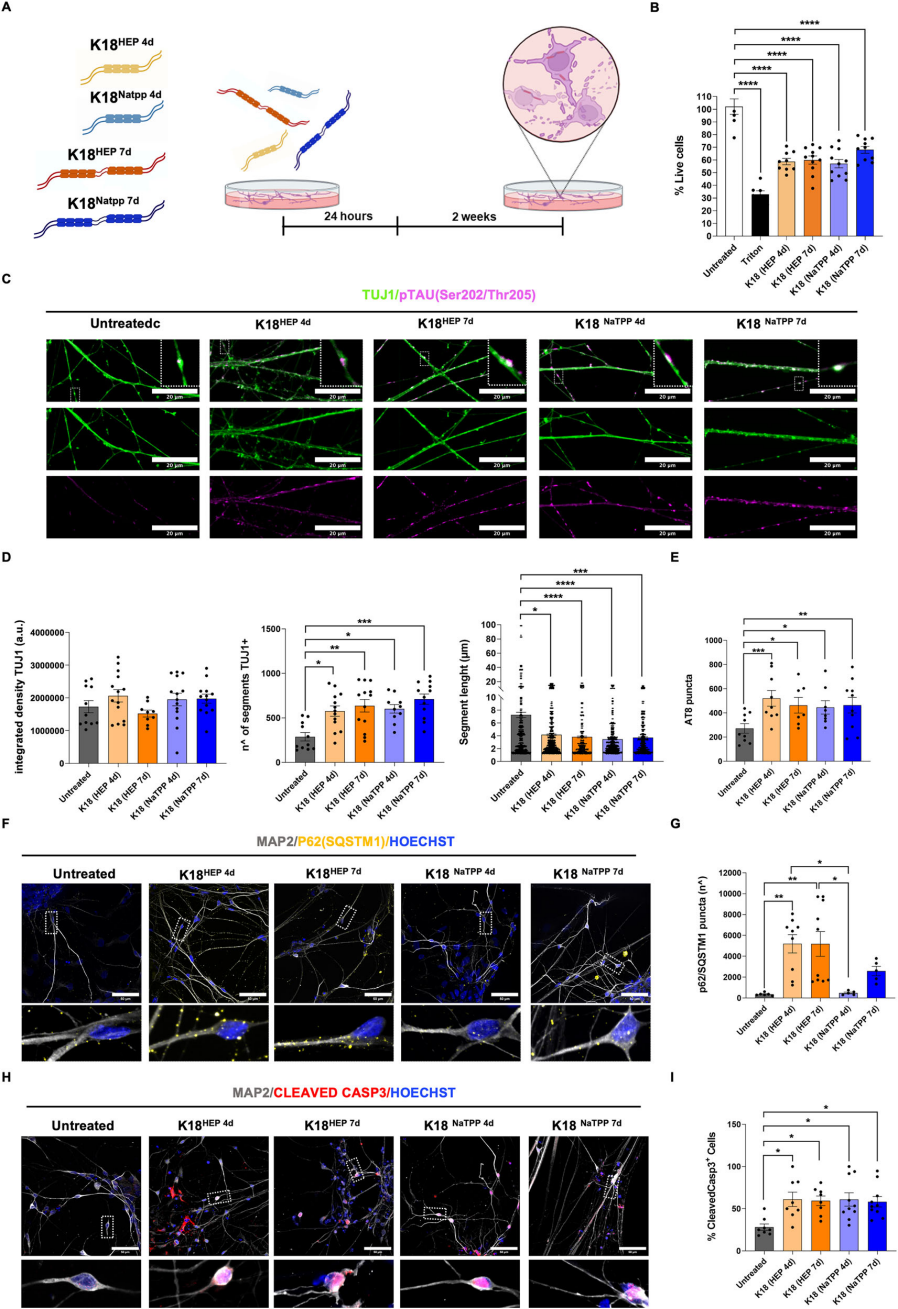
Long-term effects of tau fibrils on iPSC-derived retinal neurons. **A** Schematic representation of the experimental plan used to evaluate the long-term effect of K18 fibrils in hiPSC-derived retinal neurons. DIV 30 retinal neurons were treated with K18-Hep-4d, K18-NaTPP-4d, K18-Hep-7d, and K18-NaTPP-7d for 24 hours. Two weeks after treatment confocal microscopy experiments were performed in live imaging or after fixation Created with Biorender.com; **B** Bar charts show the effect of different K18 fibrils on cell survival. Significant differences are reported compared to the positive control (n=FOV = 9, One-way ANOVA and Tukey’s multiple comparisons post hoc test: K18-Hep-4d VS Untreated *p* < 0.0001, K18-Hep-7d VS Untreated *p* < 0.0001, K18-NaTPP-4d VS Untreated *p* < 0.0001, K18-NaTPP-7d VS Untreated *p* < 0.0001); **C** Immunostaining for neurite marker β-III-tubulin TUJ1 (green) and phospho-tau (Ser202, Thr205) (AT8, magenta) two weeks after exposure to K18 fibrils. Scale bar 20 µm; **D** Bar graphs represent the integrated signal density of TUJ1 (left panel, n=FOV = 9–14, Kruskal Wallis test and Dunn’s multiple comparisons post hoc test (ns), the number (middle panel, n=FOV = 10-13, Kruskal Wallis and Dunn’s multiple comparisons post hoc test: K18-Hep-4d VS Untreated *p* = 0.0208, K18-Hep-7d VS Untreated *p* = 0.0025, K18-NaTPP-4d VS Untreated *p* = 0.0149, K18-NaTPP-7d VS Untreated *p* = 0.0002); and the length (right panel, *n* = 103–117 segments, Kruskal Wallis and Dunn’s multiple comparisons post hoc test: K18-Hep-4d VS Untreated *p* = 0.0451, K18-Hep-7d VS Untreated *p* < 0.0001, K18-NaTPP-4d VS Untreated *p* < 0.0001, K18-NaTPP-7d VS Untreated *p* = 0.0003) of TUJ1 segments in retinal neurons; **E** Bar graph represent the puncta number of AT8 (p-tau Ser202-Thr205) (n=FOV = 15, one-way ANOVA and Sidak’s multiple comparisons post hoc test: K18-Hep-4d VS Untreated *p* = 0.0002, K18-Hep-7d VS Untreated *p* = 0.0138, K18-NaTPP-4d VS Untreated *p* = 0.0242, K18-NaTPP-7d VS Untreated *p* = 0.0046); **F** Representative confocal images of retinal neurons immunostained with p62/SQSTM1 (yellow) and neurite marker MAP2 (gray). Nuclei were stained with HOECHST (blue). Scale bar 50 µm. Zoomed images show p62 puncta within MAP2 positive neurite; **G** Quantification of p62/SQSTM1 puncta (n=FOV = 15, Kruskal Wallis and Dunn’s multiple comparisons post hoc test: K18-Hep-4d VS Untreated *p* = 0.0033, K18-Hep-7d VS Untreated *p* = 0.0019); **H** Representative confocal images of iPSC-derived retinal neurons stained with MAP2 (gray), CLEAVED-CASPASE 3 (red) and HOECHST (blue). Scale bar 50 µm; Zoomed images show cleaved-caspase 3 positive nuclei in retinal neurons; **I** Bar graph represents the percentage of cleaved-caspase 3 positive nuclei in retinal neurons, normalized to the total number of nuclei. Significant differences are reported compared to the control (n=FOV = 15, One-way ANOVA and Tukey’s multiple comparisons post hoc test: K18-Hep-4d VS Untreated *p* = 0.0174, K18-Hep-7d VS Untreated *p* = 0.0271, K18-NaTPP-4d VS Untreated *p* = 0.0115, K18-NaTPP-7d VS Untreated *p* = 0.0242).

We assessed the impact of K18 fibril treatment on neuronal dysfunction and neurodegeneration two weeks after incubation (Fig. 4A). A live-dead assay revealed a notable reduction in live cells, with a 40% decrease in cultures internalizing K18-Hep-4d and K18-Hep-7d fibrils, a 45% decrease after K18-NaTPP-4d treatment, and a 30% decrease following K18-NaTPP-7d exposure (Fig. 4B). This treatment did not alter the overall neurite staining, as indicated by TUJ1 integrated density (Fig. 4C, D, left). However, a more detailed analysis revealed that fibril-treated cultures exhibited a fragmented morphology, with a higher number of smaller TUJ1 fragments compared to untreated controls (Fig. 4D middle and right; Supplementary Fig. S8). These findings indicate that internalized fibrils gradually disrupt the neuronal cytoskeleton, with more severe effects observed in K18-NaTPP fibril-treated cultures.

In this model, fibril treatment also led to the formation of hyperphosphorylated pathological tau [15]. We measured tau phosphorylation at Ser-202, Thr-205 (AT8) epitopes (Fig. 4C) and found a significant increase in phosphorylated tau within neurites of treated cells compared to untreated ones (Fig. 4C, zoomed panel; Fig. 4E). We then explored the role of p62/SQSTM1 in processing phosphorylated tau. Immunofluorescence analysis revealed a marked increase in p62/SQSTM1 puncta in cultures treated with K18-Hep fibrils, but not in those treated with K18-NaTPP fibrils (Fig. 4F–G). Additionally, we observed a significant activation of the apoptotic pathway two weeks post-treatment, indicated by an increased Cleaved Caspase-3 signal (Fig. 4H). Additionally, nuclear analysis highlighted some abnormalities in treated retinal cultures, with reduced nuclear averaged area (Supplementary Fig. S9), thus suggesting chromatin compaction and nuclear dysfunction.

Collectively, these findings suggest that K18 fibril treatment induces long-term neurite degeneration and neuronal loss, which contribute to neurodegeneration and ultimately lead to cell death.

## Discussion

In this study, we report the different effects of K18 tau fibrils, induced by heparin and NaTPP, on human iPSC-derived retinal neurons [47–49] over short-term and long-term exposures. NaTPP and heparin impact fibrillation kinetics [28] and morphology differently, leading to distinct structural and cellular responses. Notably, NaTPP-induced fibrils showed a more rapid and extensive aggregation process compared to heparin, and produced longer, more stable fibrils over seven days. These distinctions in fibrillation dynamics translate into varied cellular effects, shedding light on the mechanisms by which different fibril structures interact with neuronal cells and contribute to neurodegeneration.

### Short-term cellular responses: tau induction and autophagy activation

Our findings suggest that K18 fibrils induce an acute stress response in retinal neurons, leading to increased expression of endogenous tau protein. This effect was most pronounced in cultures treated with K18-NaTPP-4d, which showed the highest tau upregulation among the fibril types. The selective increase in tau, confirmed via anti-tau (HT7) immunostaining, implies that exogenous fibrils may stimulate a compensatory cellular mechanism aimed at buffering against tau aggregation stress. Furthermore, K18-NaTPP-4d also caused a notable rise in autophagic flux, as indicated by the substantial increase in p62/SQSTM1 puncta, colocalized with exogenous K18 fibrils, suggesting that autophagy serves a neuroprotective role by targeting tau aggregates for degradation. Importantly, there was no evidence of Cleaved Caspase-3 activation during this short-term exposure, indicating that while K18 fibrils impose cellular stress, they do not immediately trigger apoptosis within 24 h.

### Long-term neurodegenerative effects: cytoskeleton disruption and apoptosis

Prolonged exposure to K18 fibrils led to marked neurodegenerative outcomes, highlighting the cumulative impact of sustained fibril presence on neuronal integrity. Two weeks post-treatment, we observed a significant reduction in cell viability, with the most severe decreases in cultures treated with K18-Hep-4d, K18-Hep-7d, and K18-NaTPP-4d fibrils. This decline in live cells, accompanied by neurite fragmentation, indicates that prolonged fibril internalization progressively disrupts the neuronal cytoskeleton. Furthermore, fibril-treated cells exhibited hyperphosphorylated tau at the AT8 epitope, a hallmark of tauopathy, suggesting that continuous fibril exposure fosters pathogenic tau modifications that contribute to structural degeneration.

Interestingly, long-term exposure also differentially impacted p62/SQSTM1 activity across fibril types. While K18-Hep fibrils prompted an increase in p62/SQSTM1 puncta, this was not observed with K18-NaTPP fibrils. This suggests that K18-Hep fibrils may induce a stronger autophagic response to manage phosphorylated tau, whereas K18-NaTPP fibrils may engage alternative pathways or mechanisms. The eventual activation of the apoptotic pathway, as evidenced by Cleaved Caspase-3 staining, underscores a shift from protective responses to neurodegenerative processes, marking a tipping point where cellular defenses become insufficient to counteract fibril-induced toxicity. Consistently, treated neurons displayed reduced nuclear-averaged area, indicative of chromatin compaction and nuclear abnormalities, similar to those observed in brain cortical sections from AD cases [50].

### Molecular dynamics insights: structural basis of NaTPP-induced aggregation

The K18-NaTPP-7d fibrils appear to have adopted a helicoidal mature structure, contrasting with the shorter, entangled aggregates observed in K18-Hep fibrils and K18-NaTPP-4d, which may be still in the protofibril phase [45, 52, 53]. Over time, however, both NaTPP- and Hep-induced fibrils led to tau pathology and neuronal death. In line with the hypothesis that mature fibrils, rather than intermediates, are less toxic, these differences in fibril behavior may be related to variations in their size and morphology. K18-Hep-4d fibrils were smaller and more heterogeneous, while K18-Hep-7d fibrils formed longer, more numerous structures, though their length did not exceed 400 nm. Conversely, K18-NaTPP-4d fibrils were more homogeneous and over 800 nm long, resembling the morphology of 7-day fibrils. Alongside longer polyphosphates, our results indicated that even short-chain polyphosphates like NaTPP can reduce the lag phase of fibril formation and promote elongation [43, 54]. Molecular dynamics simulations further confirmed this hypothesis. The root mean square deviation (RMSD) and radius of gyration indicated distinct conformational behaviors of Tau protein in water, phosphate buffer, and NaTPP. In this condition, K18 exhibited rapid compaction early in the simulation, a feature absent in the other systems, which likely indicates an aggregated conformation. A key finding was the behavior of the GGG patch (residues 333-335), crucial for tau fibril formation. In the K18-NaTPP system, the GGG patch remained solvent-exposed, suggesting that NaTPP stabilizes the aggregation-prone conformation of K18. Protein-ligand interactions also played a role, with specific residues (297I, 298K, 307Q, 308I, 323G) forming stable contacts with NaTPP, further stabilizing the aggregation-prone state by maintaining the exposure of the GGG patch. This hypothesis was supported by fibrillation assays and molecular dynamics simulation of single and double K18 mutants on specific positive residues, 298K and 307Q, into neutral amino acids (A). Indeed, fibrillation assays demonstrated that both K18 single mutants displayed shorter fibrils with altered morphology after NaTPP treatment. In addition, molecular dynamics simulation showed that the solvent-accessible surface area of the GGG patch in the double mutant K298A-Q307A was partially covered, reducing the binding potential, thus providing compelling evidence that the selected residues are important for NaTPP-induced fibrillation. The physiopathological relevance of NaTPP-induced tau fibrillation lies in its ability to mimic endogenous polyphosphates (polyP), which significantly influence neuronal function and excitability but also participate in pathological tau aggregation. NaTPP-induced tau fibrillation highlights the dual nature of polyP in neuronal homeostasis: while polyP modulates ion channels, regulates calcium signaling, and offers neuroprotective effects, it also promotes tau aggregation under certain conditions [27–29, 55]. This aggregation contributes to neurotoxicity by enhancing pathological tau structures associated with neurodegenerative processes, suggesting that while polyP plays a critical role in neuronal health, its impact on tau fibrillation may drive toxic outcomes in disease states.

Overall, our study demonstrated that the short-chain NaTPP plays a key role in tau folding into tangles due to molecular interactions with specific residues of K18 and in the development of pathological cellular responses in iPSC cells, providing a basis for further investigation into their potential role in neurodegenerative disease.

In this framework, it is worth mentioning that NaTPP is a key functional ingredient in food industry in meat processing by improving texture, water-holding capacity, protein solubility and gelation of myofibrillar proteins [56, 57]. It also enhances the fibrous structure of textured wheat protein, thus leading to increased protein digestibility [58]. Future investigations should also be directed to the possible substitution of NaTPP and other polyphosphates in meat and vegetables-based food products [59]. In particular, in vivo studies will be necessary to assess the presence of catalytic NaTPP concentrations in the brain.

## Supplementary information

Short-Chain Polyphosphates Induce Tau Fibrillation and Neurotoxicity in Human iPSC-Derived Retinal Neurons

## Data Availability

The datasets used and/or analyzed during the current study are available from the corresponding author upon reasonable request. For the analysis of imaging data, we utilized custom code developed in MathWorks Matlab (version 2016b), which is available upon request.

## Materials availability

All the protein structures used in this paper are available in the Protein Data Bank. The Data generated with molecular dynamics and molecular docking will be available upon request
