# Supplementary material for: Short-chain polyphosphates induce tau fibrillation and neurotoxicity in human iPSC-derived retinal neurons
Source: Cell Death Dis. 2025 May 9;16(1):369. doi: 10.1038/s41419-025-07662-5 (PMC12064648; doi:10.1038/s41419-025-07662-5)
Supplement: Supplementary file 1 — Short-Chain Polyphosphates Induce Tau Fibrillation and Neurotoxicity in Human iPSC-Derived Retinal Neurons [file 41419_2025_7662_MOESM1_ESM.docx]

**Short-Chain Polyphosphates Induce Tau Fibrillation and Neurotoxicity in Human iPSC-Derived Retinal Neurons**

*Lorenzo Barolo^1,2^*, Lorenza Mautone^3,4^*, Ylenia Gigante^2,3^*, Silvia Ghirga^2,3^, Francesco Mura^5^, Maria Vittoria Farina^1^, Stefano Tacconi^6^, Luciana Dini^6^, Giancarlo Ruocco^3,7^, Alberto Boffi^1,2,3^, Edoardo Milanetti^3,7^#, Silvia Di Angelantonio^2,3,4^#, and Paola Baiocco^1,3^#*

^1^ Department of Biochemical Sciences “Alessandro Rossi Fanelli”, Sapienza University of Rome, P.le A. Moro 5, 00185 Rome, Italy;

^2^ D-Tails srl BC, 00165 Rome, Italy.

^3^ Center for Life Nano- & Neuro-Science@Sapienza, Istituto Italiano di Tecnologia, V.le Regina Elena 291,

00161 Rome, Italy;

^4^ Department of Physiology and Pharmacology, Sapienza University of Rome, 00185 Rome, Italy

^5^ Research Center on Nanotechnologies Applied to Engineering of Sapienza (CNIS), Sapienza University of Rome, 00185, Rome, Italy.

^6^ Department of Biology and Biotechnologies, Sapienza University of Rome, 00185 Rome, Italy

^7^ Department of Physics, Sapienza University of Rome, 00185 Rome, Italy

* L.B., L.M. and Y.G. These authors contributed equally to this work

# E.M., S.D.A. and P.B. These authors contributed equally to this work

Correspondence to: [silvia.diangelantonio@uniroma1.it](mailto:silvia.diangelantonio@uniroma1.it), [paola.baiocco@uniroma1.it](mailto:paola.baiocco@uniroma1.it)

**Running title: Polyphosphates induce neurotoxic tau fibrils**

**SUPPLEMENTARY MATERIAL AND METHODS**

**Purification of K18 domain and mutants.**

The genes coding for K18 WT, mutant K298A, mutant Q307A, and double mutant K298AQ307A were cloned into a pET30a vector and inserted into Escherichia coli cells. The K18 domain of tau protein and mutants were expressed, purified, and biochemically characterized as described (1), (2). Briefly, to extract the protein, 20 g of harvested cells were resuspended in 100 mL of lysis buffer (20mM MES, 1mM EDTA, 0,2 mM MgCl_2_, 300 mM NaCl, protease inhibitor, and DNAse, pH 6.4). Lysis was carried at 50% amplitude for 30 minutes, 3 seconds ON and 6 seconds OFF on ice. The lysate was centrifuged at 15000 g for 30 minutes at 4°C. The supernatant was separated from the cell debris and was subsequently heated at 80°C for 20 minutes, to promote the precipitation of a large amount of undesired *E. coli* proteins. After heating, the sample was centrifuged at 15000 g for 30 minutes at 4°C. The precipitated proteins were separated and the supernatant was left in a 2 L solution of dialysis buffer (20mM MES, 50mM NaCl, 1mM EDTA, 1mM MgCl_2_, pH 6.4) overnight at 4°C. The recombinant K18 and its mutants were purified using a two-step chromatography. The first step was a cation exchange chromatography, using a HiPrep™ SP HP 16/10 column (Cytiva, Marlborough, UK) mounted on an ÄKTA® Pure (Cytiva) chromatograph. Buffer A (20 mM MES, 1mM EDTA, 1 mM MgCl_2_, 50 mM NaCl), Buffer B (20mM MES, 1mM EDTA, 1 mM MgCl_2_, 1 M NaCl). For K18 WT, the method was: 2 CV 10% Buffer B, 2 CV 15% Buffer B, 2 CV 20% Buffer B, 2 CV 25% Buffer B, 2 CV 30% Buffer B, 2 CV 50% Buffer B, and 2 CV 100% Buffer B. For the three mutants, the method was slightly different: 2 CV 15% Buffer B, 2 CV 30% Buffer B, and 2 CV 100% Buffer B. Based on the final chromatogram, the elution fractions were analyzed via gel electrophoresis. Mutant K298A and double mutant K298AQ307A reported a high percentage of proteolyzed protein after purification. To avoid degradation, the heating step was removed for these two proteins. However, the double mutant still reported auto-proteolysis, therefore the analysis resulted impossible for the K298AQ307A protein. The fractions containing the protein of interest were selected and prepared for the second chromatographic step, size exclusion chromatography. The selected fractions were purified using a HiLoad™ 26/600 Superdex 75 (Cytiva). The final concentration of tau K18 WT was determined via UV-Vis spectrophotometry using Jasco V-750 (Jasco Corp., Tokyo, Japan). The molar extinction coefficient ε_270nm_, for K18, is 1490 M^-1^ cm^-1^.

**Formation and fluorescence analysis of K18 fibrils**

K18 WT, K18 K298A, and K18 Q307A were all prepared for fibrillation using the same method. 100 µM of protein in PBS was reduced using 1 mM TCEP 1 mM (Tris(2-carboxyethyl) phosphinehydrochloride), for 10 minutes at 55°C. The reduced protein was subjected to aggregation agents. K18 WT was exposed to either sodium heparin (Sigma) or NaTPP (Sigma) added in ratio 1:1 (100 µM) to initiate tau protein aggregation *in vitro*. K18 K298A and K18 Q307A were subjected only to NaTPP. The samples were kept at 37°C under rotation at 150 rpm for several days. The K18 WT samples exposed to heparin will be referred to as K18-Hep, whilst the ones subjected to NaTPP will be called K18-NaTPP. Multiple time points of K18-Hep and K18-NaTPP, both in the ratio 1:1 = protein: cofactor, were collected to monitor the fibrillation of the samples. The chosen time points were as follows: 0, 1, 3, 4, 7, 10, 14 days. The fibrillation status of the protein was reported using fluorescence analysis. Firstly, the fibrillation kinetics were analyzed using a well-known fluorophore, thioflavin T (ThT) (3). However, due to the limited specificity of ThT towards K18 fibrils and the unexpected inertness of certain phosphates in generating ThT signals, we opted for the BODIPY-based probe BT1, loaded into humanized ferritin nanocages to enhance the solubilization of the probe and its fluorescent signal (4), (5). A 1 mg/mL concentration of HumAfFt-BT1 was added to 400 µL of each K18 sample. Fluorescence was measured using excitation at 530 nm and emission at 565 nm with an Excitation bandwidth of 5 and emission bandwidth of 10 nm. All the measurements were made by using the RF-6000 fluorimeter (Shimadzu RF-6000). The mutants K298A and Q307A exposed to NaTPP were analyzed only with HumAfFt-BT1.

**Scanning transmission electron microscopy (STEM) of K18 fibrils**

STEM images of fibrillated K18 WT, K18 K298A, and K18 Q307A were produced and analyzed. The samples were kept at 37°C for 4 and 7 days under rotation at 150 rpm. One sample was not incubated at 37°C and was used as negative control. The samples were prepared for STEM imaging. The samples were diluted with H_2_O from 100 µM to 50 µM. One drop of sample was loaded onto a carbon-coated 3 mm copper grid. The sample was left to adsorb for 5 minutes. The grid was washed with one drop of water for 5 minutes. Then, one drop of the staining solution containing 3% uranyl acetate was added, and the mixture was incubated for 5 minutes. The grid was washed twice with H_2_O. All the samples were visualized via a Zeiss Auriga microscope in STEM mode. ImageJ software was used to calculate the length and population distribution of the fibrils. For population distribution, the automatic particle counting tool of ImageJ removed the background with a manually selected threshold and calculated the percentage of space occupied by fibrils in the STEM image (mean n=3).

**Computational Method**

The protein (and if present also the small molecule) was placed in a dodecahedron simulative box, with periodic boundary conditions, filled with TIP3P water molecules (6). Each protein atom was at least at a distance of 1.1 nm from the box borders. The system was then minimized with the steepest descent algorithm. Next, a relaxation of water molecules and thermalization of the system was run in NVT and NPT environments each for 0.1 ns at 2 fs time-step. The temperature was kept constant at 300 K with the v-rescale algorithm (7); the final pressure was fixed at 1 bar with the Parrinello-Rahman algorithm (8). LINCS algorithm (9)was used to constrain h-bonds. A cut-off of 12 ̊A was imposed to evaluate short-range non-bonded interactions and the Particle Mesh Ewald method (10) for the long-range electrostatic interactions. Finally, we performed one microsecond of molecular dynamics for each molecular system with a time step of 2 fs, saving configurations every 2 ps. On each frame of the trajectory (for a total of 1000 snapshots per simulation), we use the DMS method (11) to compute the solvent-accessible surface, using a density of 5 points per Å and a water probe radius of 1.4 Å.

**Retinal neuron differentiation from human iPSC.**

Human iPSC (SIGi001-A, EBiSC/Sigma) were differentiated into retinal neurons following a multi-step protocol (12), (13)with minor modifications. Human iPSCs were maintained in mTeSR Plus and dissociated into single cells with Accutase (Gibco) once the colonies reached ~70% confluency. Dissociated hiPSC were plated on growth factor reduced Matrigel coated dishes (Corning, dilution 1:100) at a density of 1000 cells/mm^2^. This day was defined as day in vitro (DIV) -2. The next day, the medium was completely replaced with N2B27 medium (50% DMEM/F12, Sigma; 50% Neurobasal, ThermoFisher; 1% GlutaMAX Supplement, Gibco; 0.1% Pen-Strep, Sigma; 1% NEEA, Gibco; 1% N2 Supplement, ThermoFisher; 2% B27 Supplement w/oA, Gibco) and refreshed daily. Reaching day 20 of differentiation (DIV 20), cells were dissociated with Accutase and plated onto PLO/Laminin coated cover glasses circle (0.12 mm, ThorLab) at a density of 100.000 cells per glass until DIV 30. A different mix of small molecules was added to the medium at specific intervals: 1 µM Dorsomorphin (Sigma) and 2.5 µM IDE2 (Sigma) from DIV 0 to DIV 6; 10 mM Nicotinamide (Sigma) until DIV 10; 25 µM Forskolin (Sigma) from DIV 0 to DIV 30; 10 µM DAPT (Prepotech) from DIV 20 to DIV 30. For subsequent analysis, at least three different experiments (biological replicates) were conducted. DIV 30 retinal cultures were treated with 5 µM of K18-Heparin (4 and 7 days) and K18-NaTPP (4 and 7 days) for 24 hours at 37°C in 5% CO_2_. The effects of fibrils were evaluated 24 hours and two weeks after K18 treatment.

**Assessment of rhodamine-labeled K18 fibrils into cultured iPSC-derived retinal neurons.**

Rhodamine-labeled K18 samples were prepared by reducing 100 µM K18 in PBS using TCEP 1 mM, for 10 minutes at 55°C. Subsequently, 500 µM of the fluorescent dye tetramethylrhodamine-5-maleimide (Sigma-Aldrich) was added to the protein. The solution was kept for 2 hours at room temperature. To remove all the unbounded rhodamine, the solution was purified using a MidiTrap G25 desalting column equilibrated in PBS (Cytiva). UV-Vis measurements measured the labeling percentage using a molar excitation coefficient ε_555nm_ = 90000 M^-1^cm^-1^. The final rhodamine labeling percentage was 65 ± 5%. Rhodamine-labeled K18 was incubated at 37°C in heparin 100 µM or NaTPP 100 µM. Fibrillation of the fluorescent-labeled K18 was confirmed by STEM microscopy (Supplementary Fig. S1). DIV 30 retinal neurons were treated with the rhodamine-labeled fibrillated K18 samples for 1 hour, 24 hours, and 48 hours. Neurons were stained with a staining solution of Fluorescein Diacetate (FDA, 8 µg/ml, Sigma) and Hoechst (3µg/ml, Sigma). Live images were acquired with an air objective 20X/NA 0.42 (Olympus) on an Olympus iX73 microscope equipped with an X-Light V3 spinning disc head (CrestOptics), an LDI laser illuminator (89 North), a PRIME cMOS camera and a MetaMorph software (Molecular Devices). Images were stacked in maximum Z-projections and the assessment of K18-uptake was performed using the ImageJ software (https://imagej.net/ij). To quantify the amount of cellular internalization of K18, FDA signals were used to delineate neurons, and the area covered by rhodamine-fluorescent spots inside neurons was reported. The analysis comprised two main steps: in the initial step, a mask was generated based on the FDA signal using a global thresholding method; then, the mask was transferred to the rhodamine channel and the area covered by rhodamine in neurons was calculated and normalized on the total number of cells in the field of view (FOV).

**Cytotoxicity analysis of tau fibrils**

Fluorescent-based live-dead assay was performed using 8 µg/ml of Fluorescein Diacetate (FDA) to stain live cells, 20 µg/ml Propidium Iodide (PI) to stain dead cell and Hoechst (1:300) to stain nuclei. The cells were incubated with K18-Heparin (4 and 7 days) and with K18-NaTPP (4 and 7 days) for 24 hours. The day after, neurons were stained with a staining solution for 5 minutes in the dark. Round cover glasses with neurons were transferred into an Ibidi glass bottom dish in HEPES-buffered external solution (NES) containing 140 mM NaCl, 2.8 mM KCl, 2 mM CaCl_2_, 2 mM MgCl_2_, 10 mM HEPES, 10 mM D-glucose (pH 7.3 with NaOH; 290 mOsm). The live/dead assay was performed after 24 hours and two weeks after the initial seeding of K18. Live imaging was performed with an air objective 20X/NA 0.42 (Olympus) on an Olympus iX73 microscope equipped with an X-Light V3 spinning disc head (CrestOptics), an LDI laser illuminator (89 North), a PRIME cMOS camera and a MetaMorph software (Molecular Devices). Images were stacked in maximum Z-projections and the counting of the cells was conducted using ImageJ software. The live cells were counted in the FDA channel and the percentage was calculated as (Live Cells/Total Cells Number) * 100. The dead cells were counted in the PI channel and their percentage was calculated as (Dead Cells/Total Cells Number) * 100.

**Immunostaining and confocal image acquisition.**

iPSC-derived retinal neurons were fixed with 4% paraformaldehyde (PFA, Sigma Aldrich) at DIV 30 of differentiation protocol. The immunostaining was performed after 24 hours and after 2 weeks of the initial seeding (K18-Hep 4 and 7 days, K18-NaTPP 4 and 7 days). The fixed neurons were permeabilized using a PBS solution containing 0.2% Triton X-100 (Merck Life Science) for 10 min and, subsequently, were incubated with a blocking solution containing PBS 0.1% Tween-20 (Sigma-Aldrich) and 5% goat serum (Merck KGaA, Darmstadt, Germany) for 45 minutes at room temperature. Then, the cells were incubated with the primary antibodies overnight at 4°C. The primary antibodies used are TUJ1 (1:1000 rabbit, T2200, Sigma), pTAU Ser202/Ser 205 (1:200, mouse, MN1020, Invitrogen), HT7 (1:1000 mouse, MN1000, Invitrogen), p62/SQSTM1 (1:200, mouse, 56416, Abcam), MAP2 (1:2000 chicken, ab5392, Abcam), Cleaved-caspase 3 (1:500, rabbit, 9661s, Cell signaling). The day after, the cells were incubated with AlexaFluor secondary antibodies (goat anti-rabbit, goat anti-mouse, goat anti-chicken, 1:750, ThermoFisher Scientific) for 1 hour at room temperature and then with PBS solution containing 0.1% Tween-20, adding Hoechst (1:300, 33258, Merck) to stain nuclei. Control experiments in the absence of primary antibody incubation were performed to check staining specificity. Confocal stack images (2048 x 2048 pixels) were acquired on an Olympus iX73 microscope equipped with an X-Light V3 spinning disc head (CrestOptics), an LDI laser illuminator (89 North), a PRIME cMOS camera and a MetaMorph software (Molecular Devices). The imaging was performed with an oil immersion objective of 60× magnification, and a numerical aperture (NA) of 1.42 (Olympus).

**Immunofluorescence analysis of neurites, autophagic and apoptotic cells.**

The analysis was conducted on iPSC-derived retinal neurons 24 hours and two weeks after K18 treatment. Images were stacked in maximum Z-projections and analyzed using the ImageJ software. The background has been removed from all images.

The cytoskeleton analysis was performed by measuring the % of area covered by β-III Tubulin (TUJ1) and MAP2. To enhance the assessment of cytoskeletal integrity in response to K18 treatment, the analysis was implemented by incorporating a binarization step for the TUJ1 channel using a thresholding method (Otsu). After binarization, the images were segmented (Fig. S7A). The identification of TUJ1-positive segments was achieved through the "find maxima" function in ImageJ (with parameter prominence > 500). Lastly, the length of these segments was determined using the "analyze particles" function in ImageJ (with parameter size pixel^2=100-infinity).

The quantification of total tau was achieved by measuring the % of area covered by the HT7 signal. Phospho-Tau Ser202/Ser 205 levels were measured using the "find maxima" function in ImageJ (with parameter: prominence > 50).

The p62/SQSTM1 signal was analyzed through the “find maxima” function in ImageJ (with parameter prominence >50) and reported as puncta. For colocalization analysis, Mander’s overlap coefficient was evaluated with the Jacob plugin in ImageJ. Mander’s coefficient quantifies the co-occurrence of rhodamine-K18 with the antibody p62/SQSTM1 as the fraction of the antibody signal coinciding with the K18 in the binarized images.

The cleaved-caspase 3 analysis was performed counting manually the cleaved-caspase 3 positive nuclei. The percentage of the cleaved-caspase 3 positive nuclei was calculated as (cleaved-caspase 3 cells/total cells number) * 100.

**SUPPLEMENTARY FIGURES**

**Supplementary Figure S1**

**
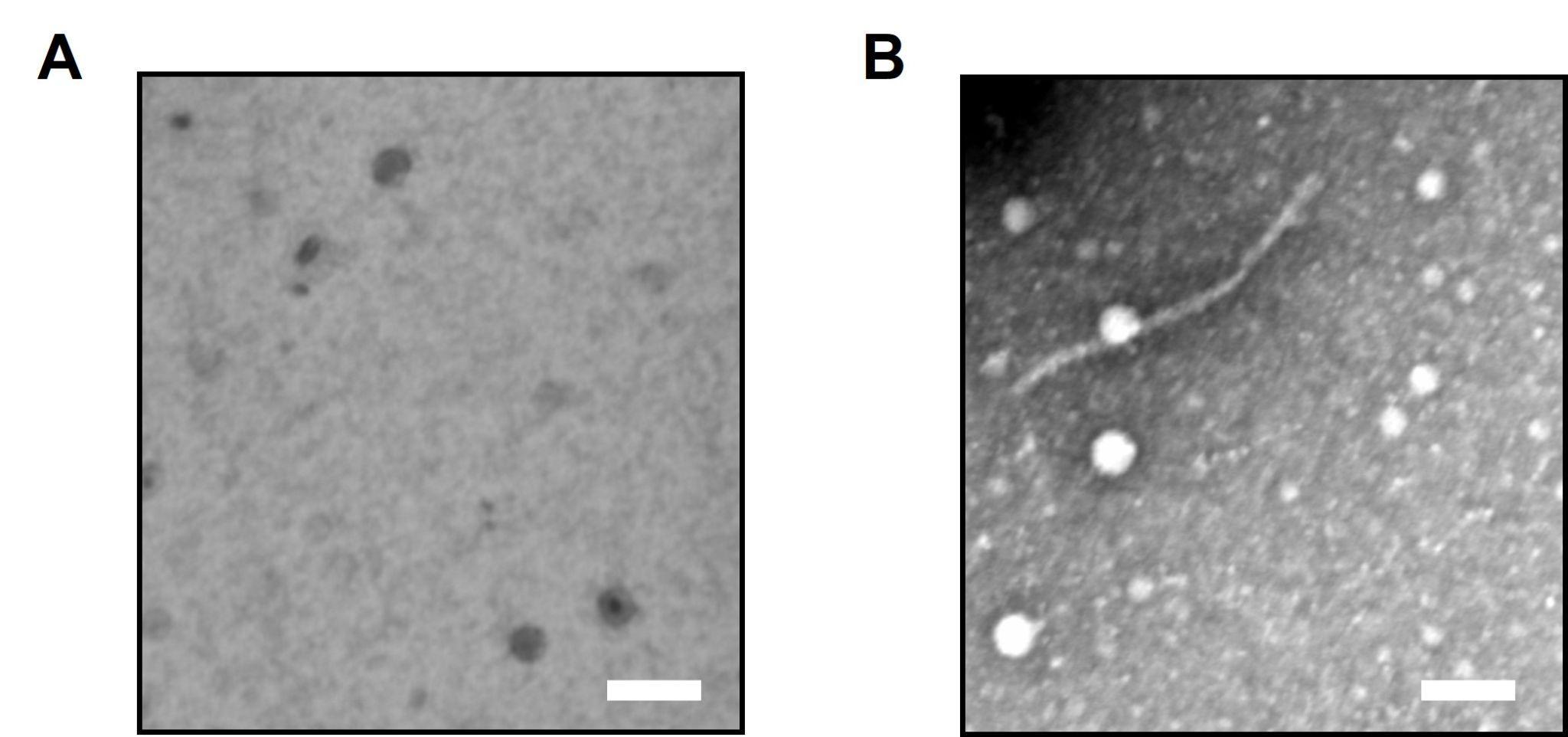
**

**Supplementary Figure S1**: Representative STEM images of rod-K18 A) CTRL and B) fibrillated for 7 days at 37°C. The scale bar is 100 nm.

**Supplementary Figure S2**


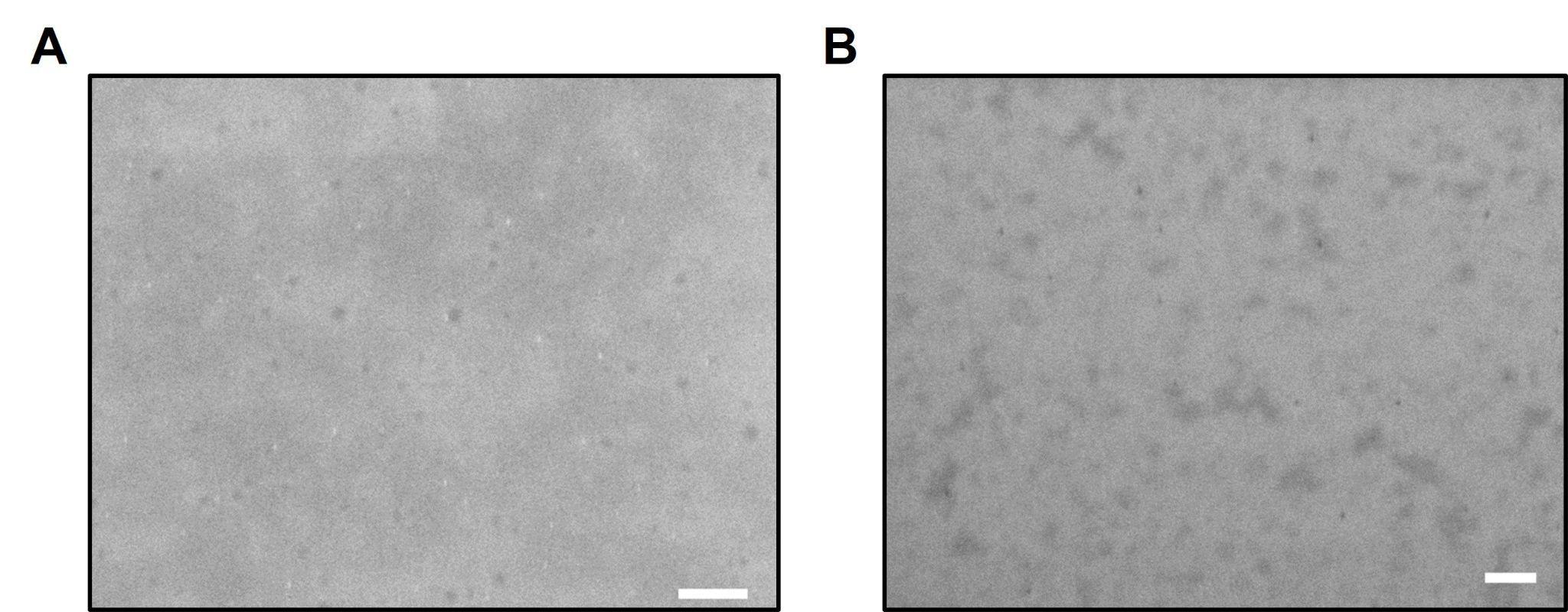


**Supplementary Figure S2:** Representative STEM images of K18 in sodium monophosphate (NaP) incubated at 37°C without any aggregation agent. The scale bars are 200 and 100 nm in A and B respectively.

**Supplementary Figure S3**


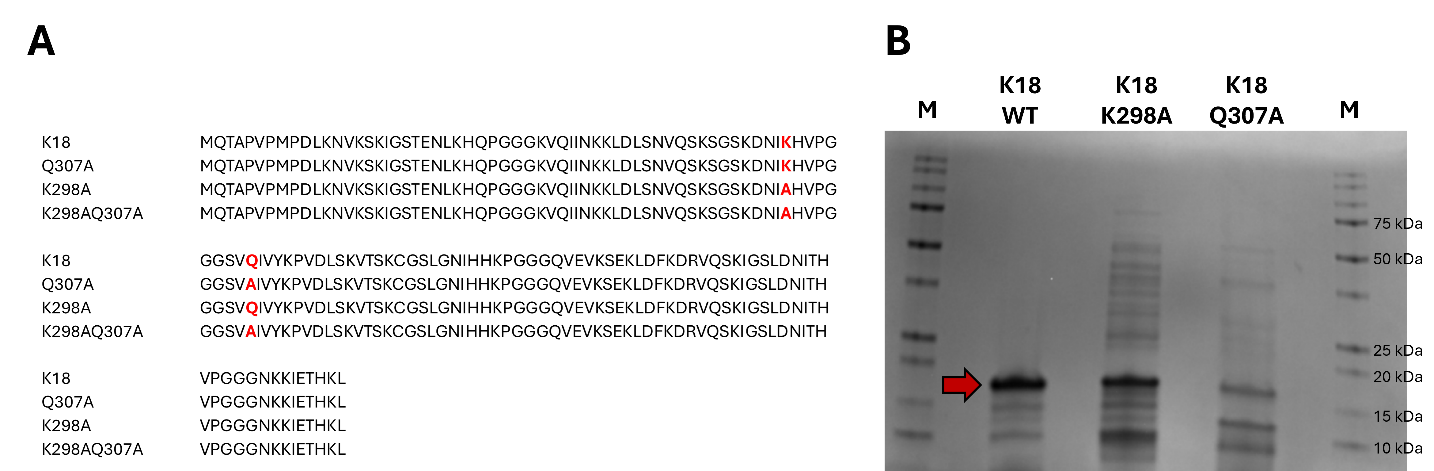


**Supplementary Figure S3:** **A)** Aminoacidic sequence of K18 domain and mutants. The mutations are shown in red. **B)** SDS-PAGE of K18 WT and mutants after the purification procedure described above.

**Supplementary results and Figure S4**

**Supplementary analysis of molecular dynamics simulation data**

A preliminary analysis of the Root Mean Square Deviation (RMSD) for all three systems showed a significant deviation from the initial structure (Fig. 2A). This process was much more rapid for the system with NaTPP (in orange), significantly slower for the free form of K18 (in gray), and intermediate for the system containing NaP (in green). Interestingly, we observed that the form containing NaTPP had an RMSD standard deviation of 0.23 nm, a markedly lower value than the other two systems, which were 0.36 nm and 0.38 nm for the NaP system and the free form, respectively. This indicates that the conformational space explored by K18 in the presence of NaTPP was much more limited than those explored in the other two systems. An analysis of contacts showed the interaction between K18 and the ligand, involving some residues of the protein in the binding. As shown in the cartoon representation in Fig. 2B, we observed compaction of the protein when it interacted with the ligand (only the NaTPP case is shown in Fig. 2B). In addition, the radius of gyration for each time of the three systems was calculated (Fig. 2C, in orange After about 20 ns, K18 interacting with NaTPP underwent compaction, which remained, albeit with evident structural fluctuations, throughout the simulation. In the system containing the NaP ligand, we also observed compaction of the K18 protein, although this process occurred after the first 100 ns (Fig. 2C, in green). The radius of gyration trend for the free form in solution (Fig. 2C, in grey) fluctuated more in the first half, transitioning from more compact conformations to more disordered and extended ones. Since all three conditions led to a compact state of the protein in the simulation (low radius of gyration, between 1 and 2 nm), it is not straightforward to attribute the causes of aggregate formation to a possible folding process of the protein in the presence of a specific ligand. The standard deviation of the radius of gyration values was significantly smaller (0.28 nm) for the K18-NaTPP molecular system compared to the other two systems (0.67 nm and 0.81 nm for K18-NaP and the free form, respectively), indicating that the conformations explored by the K18-NaTPP complex were more stable compared to the other two simulated systems (Fig. 2C).

An additional molecular dynamics simulation of 1 microsecond was performed following the mutation of the structure, where the amino acids of the two aforementioned residues were replaced with alanine, namely K298A-Q307A. The simulation was conducted using the same protocol as the other simulations. In particular, to assess solely the effect of the proposed mutations and thus the role of the selected residues in the structural rearrangement of K18, the simulation was performed in the presence of triphosphate. The comparison between the two simulated systems was carried out by evaluating the solvent-accessible molecular surface area of the three residues (GGG) involved in the tau fibril interface. Although the mutated form also exhibits stabilization of the solvent-exposed surface of the selected patch (evident from the similarity in the standard deviation of the surface area distributions of the selected residues) the double-mutant form shows a significantly smaller solvent exposure of the patch involved in the Tau fibril interface compared to the non-mutated form. The distribution of the solvent-exposed area of the three GGG residues in the mutated form is shifted toward lower values compared to the WT form. In fact, the mean values, both for the equilibrium region alone (121 Å² and 109 Å² for the WT and mutated form, respectively) and for the entire simulation (117 Å² and 106 Å² for the WT and mutated form, respectively), are notably different. The Kolmogorov-Smirnov test confirms that the two distributions are shifted in both cases with a p-value less than 0.05, suggesting that the reduced solvent exposure of the same patch in the mutated form could be the cause of the failed assembly.

**
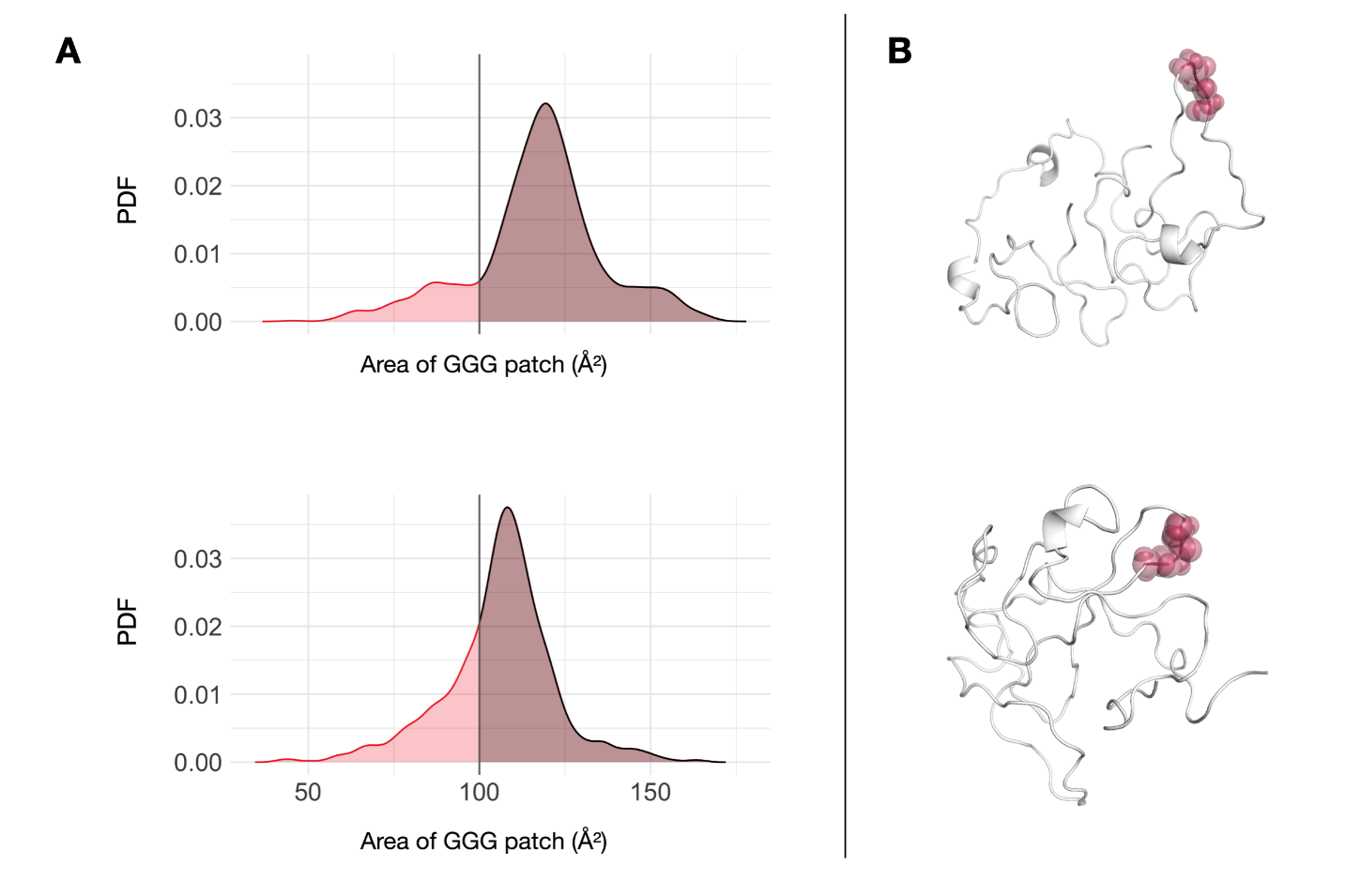
**

**Supplementary Figure S4:** Analysis of Solvent Exposure of the Patch Involved in the tau Fibril Interface for Both the WT and the Double Mutation Form. **A)** Distribution of solvent-exposed surface area values for the residues involved in the interface of the experimental tau fibril structure (fragment GGG). The solvent-exposed surface area values for the three residues of the GGG fragment were calculated for each frame of the simulation, considering one conformation every 1 ns. The probability density function (PDF) for both the WT form (top) and the mutated form (bottom) is shown. A vertical line at 100 Å² is shown to better emphasize the shift of the two distributions. **B)** Two structures randomly extracted from the ensemble of configurations explored during molecular dynamics simulations. The residues corresponding to the GGG fragment are highlighted in red.

**Supplementary Results and Figure S5**

Purified K18-K298A and K18-Q307A mutants (100 μM) were exposed to NaTPP 100 μM for 7 days at 37°C and the formation of tau-based β-sheet structures was analyzed by fluorescence measurements using the HumAfFt-BT1 (λem=565 nm), the same used for the WT. The mutation of either K298 or Q307 showed a modification of K18 fibrillation, confirming our thesis of a possible interaction between the positively charged amino acids and the negatively charged tripolyphosphate. As shown in figure S8A, the relative fluorescence after 7 days of fibrillation was almost the same as the control for mutant K298A. On the other hand, for mutant Q308A, an increase in fluorescence was noticeable. Interestingly, the STEM images showed the presence of fibrils for both K298A and Q308A (Fig. S4 C-D). However, the shape and length of the fibrils were different compared to the WT (Fig. S4 B), confirming that such mutations alter the fibrillation process mainly on lysine site as reported elsewhere (14).

**
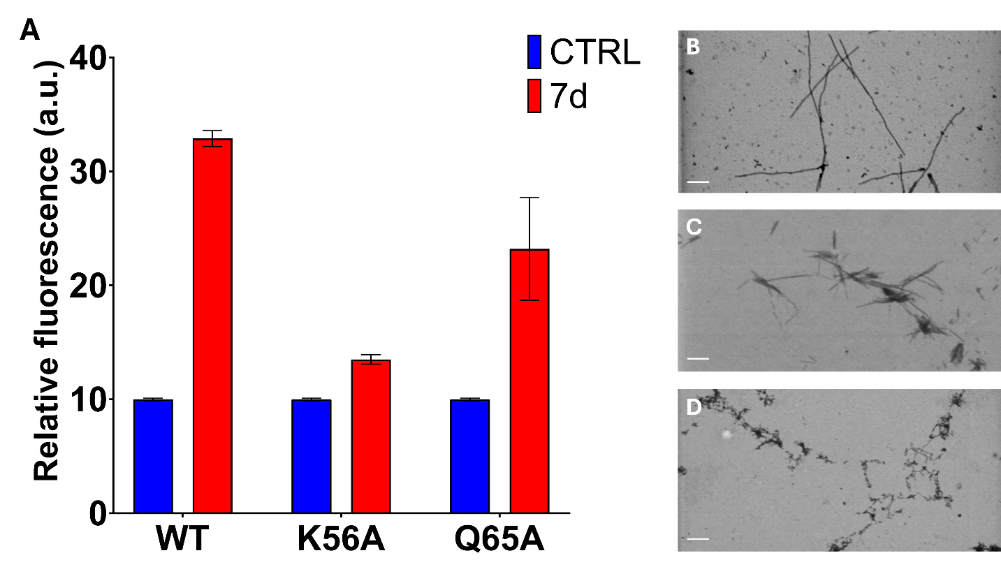
**

**Supplementary Figure S5:** **A)** Representative plot showing the fibril formation of K18 WT, K18 K298A, and K18 Q307A using BT1 fluorescence as a function of time in the presence of a non-fibrillated control for each protein, 100 µM, and protein fibrillated after exposure to NaTPP 100 µM (red bar) at 37°C at different time points. All spectra were obtained using a λex = 530 nm, λem = 565 nm. All data are mean ± SEM, n=3. On the right panel, a comparison of representative STEM images of **B)** K18wt + NaTPP **C)** K18-K298A + NaTTP and **D)** K18-Q307A + NaTPP incubated at 37°C for 7 days. The scale bars are 200 nm.

**Supplementary Results and Figure S6**

**NaTPP-Induced K18 Fibrils Internalization and Cytotoxic Effects in Human iPSC-Derived Retinal Neurons**

To visualize the cellular uptake of K18 fibrils, we conjugated tetramethyl-rhodamine to K18 following typical fibrillogenesis with heparin and NaTPP, as described in the method section resulting in a 65 ± 5% of rhodamine labeling per protein concentration. The rhodamine-labeled K18 fibrils formation was assessed by the acquisition of STEM images (Supplementary Fig. S2). Afterward, DIV 30 human iPSC-derived retinal neurons were incubated with 5 µM of different preparation of rhodamine-labeled K18 fibrils for 1 hour (15) and 24 hours (16), (17) at 37°C and 5% CO_2_ (Supplementary Fig. S3A). Immediately after, neurons were stained with fluorescein diacetate (FDA) and Hoechst for nuclei visualization and acquired with a confocal microscope. The fluorescence of rhodamine was measured at each time point. Live-cell imaging revealed a progressive enhancement in rhodamine intracellular fluorescence over time (Supplementary Fig. S3C) reflecting the time-dependent uptake of both K18-Hep and K18-NaTPP fibrils (Supplementary Fig. S3B), but their combined effect is not significant. We consistently observed the same fluorescence intensities across all conditions after 24-hour treatment (Supplementary Fig. S3B). This result suggests that 24-hour treatment is sufficient for both K18-Hep and K18-NaTPP exogenous fibrils to be internalized in DIV 30 hiPSC-derived retinal neurons. Next, we investigated the short-term effects of K18 fibrils on neuronal viability. We treated for 24 hours retinal neurons with 5 µM of K18-Hep-4d, K18-Hep-7d, K18-NaTPP-4d, K18-NaTPP-7d (Supplementary Fig. S3D). Initially, we performed a live-dead assay with fluorescein diacetate (FDA), a live-cell dye, and propidium iodide, an indicator of cell death. At a concentration of 5 µM K18 fibrils, 24 hours after exposure, hiPSC-retinal neurons treated with K18-Hep-4d exhibited a 40% decrease in live cells compared to untreated cells. Similarly, retinal neurons treated with K18-Hep-7d fibrils exhibited a 30% reduction in live cells count compared to untreated cells. In addition, there was a 20% reduction in live cells after 24 hour exposure to K18-NaTPP-4d and K18-NaTPP-7d fibrils, compared to the untreated cells (Supplementary Fig. S3E).


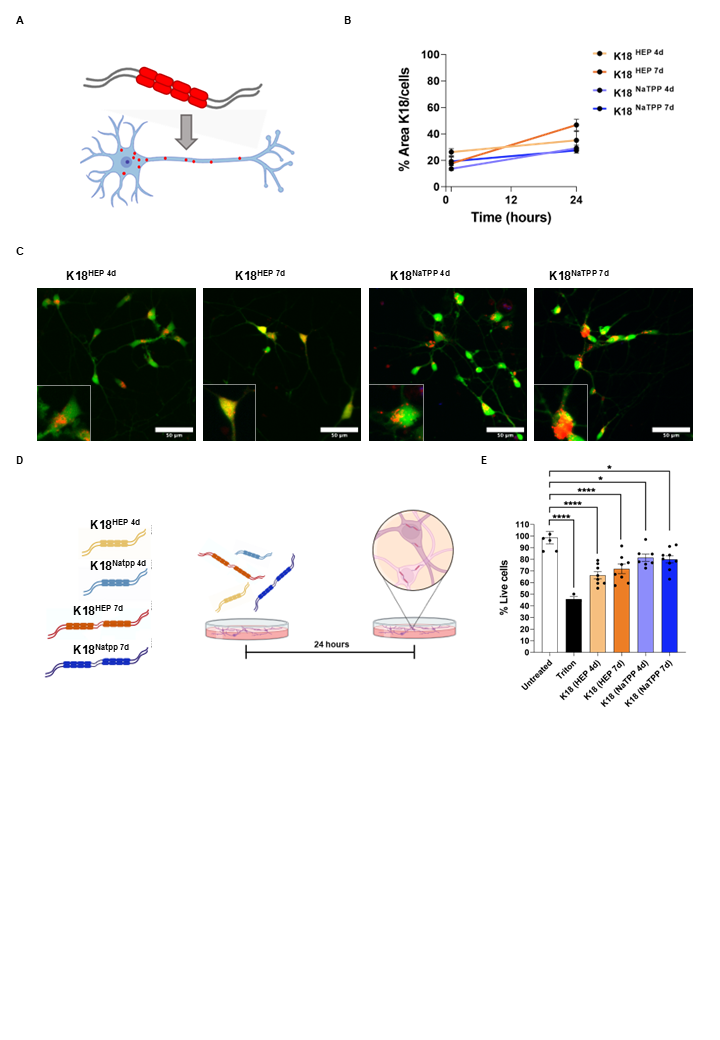


**Supplementary Figure S6**: **A)** Schematic representation of rhodamine-labeled K18 fibrils formation and their internalization in neurons. **B)** Time course of the internalization of 5 µM of rhodamine-labeled K18 samples after 1 and 24 hours of incubation, normalized on the total number of cells in the FOV (field of view). (n=FOV=4, two-way ANOVA factor 1=time p<0.0001, factor 2=treatment p=0.0427 and Sidak's multiple comparisons post hoc tests); **C)** Uptake of 5 μM preformed rhodamine-labeled K18 samples after 24 h (red) in human iPSC-derived retinal neurons stained with fluorescein diacetate (green). Scale bar 50 µm. Zoomed images show the cytoplasmic distribution of rhodamine-labeled K18 samples; **D)** Schematic representation of the experimental plan used to evaluate the effect of K18-tau fibrils in hiPSC-derived retinal neurons (DIV 30). Neurons were treated with k18-fibrils (K18-hep4-d, k18-hep-7d, K18-NaTPP-4-d, K18-NaTPP-7-d) for 24 hours and then confocal microscopy experiments were performed in live-imaging or after fixation; **E)** Bar chart shows the effect on cells survival after each treatment. Significant differences are reported compared to positive control. Black dot represents a single field of view (FOV) (n=FOV=9, One-way ANOVA p<0.0001 and Holm-Sidak's multiple comparisons post hoc tests: K18-Hep-4d VS Untreated p=0.0007; K18-Hep-7d VS Untreated p=0.0063; K18-NaTPP-4d VS Untreated p=0.0131; K18-NaTPP-7d VS Untreated p=0.0159).

**Supplementary Figure S7**

**
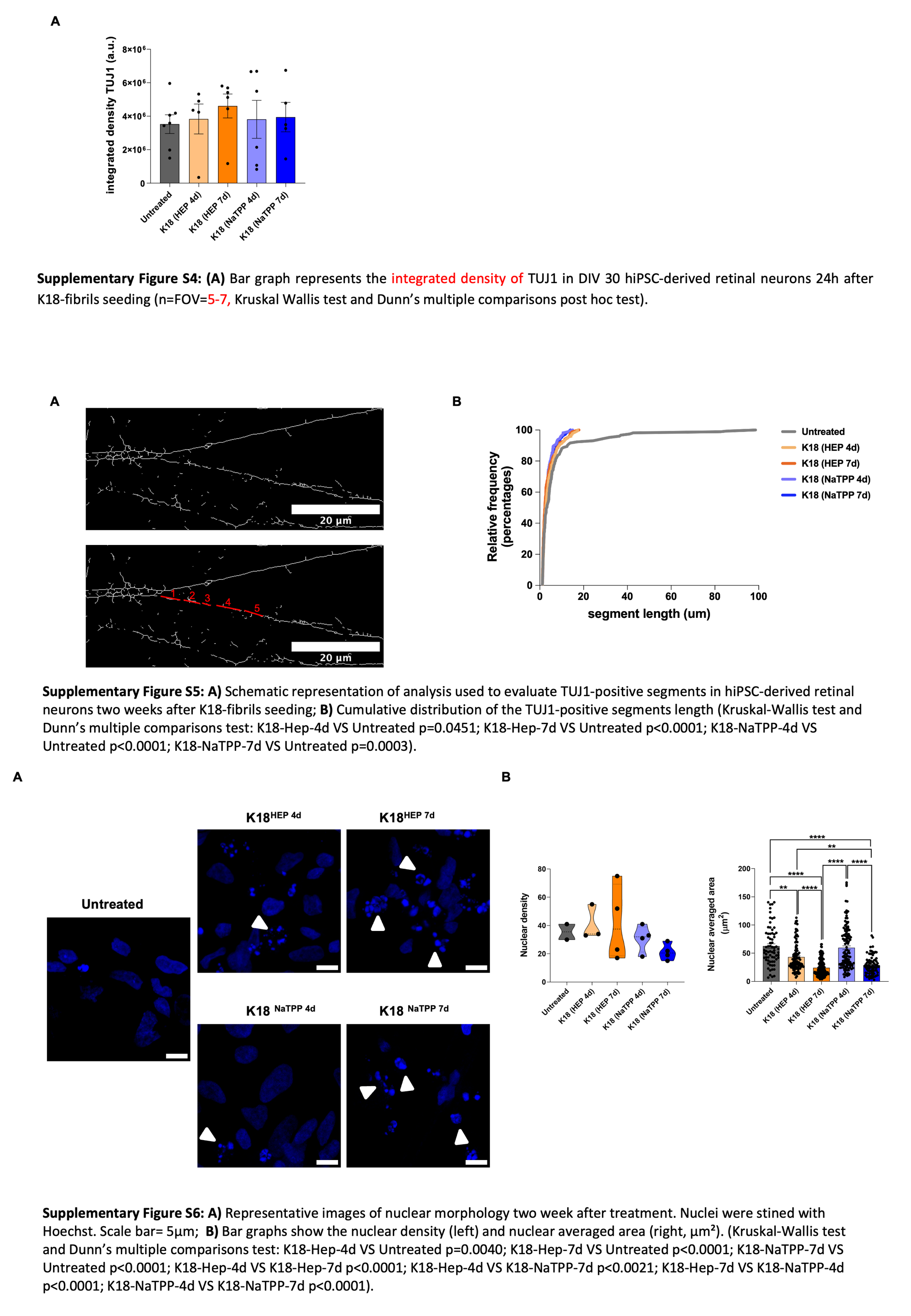
**

**Supplementary Figure S7: (A)** Bar graph represents the integrated signal density of TUJ1 in DIV 30 hiPSC-derived retinal neurons 24h after K18-fibrils seeding (n=FOV=9, Kruskal Wallis test and Dunn’s multiple comparisons post hoc test).

**Supplementary Figure S8**


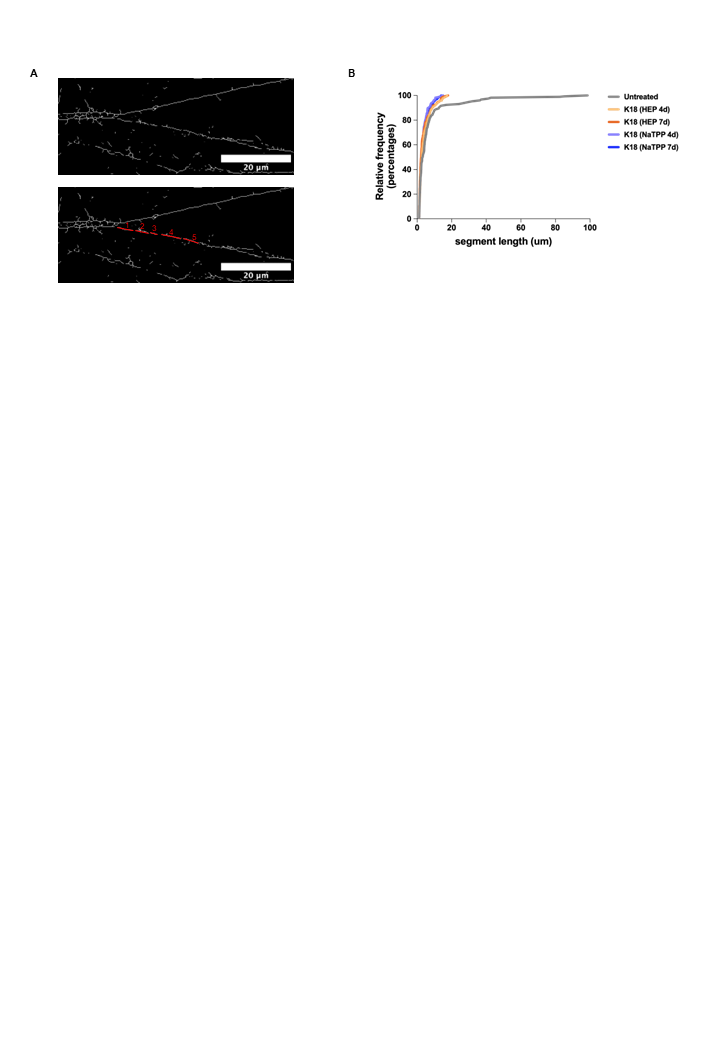


**Supplementary Figure S8:** **(A)** Schematic representation of analysis used to evaluate TUJ1-positive segments in hiPSC-derived retinal neurons two weeks after K18-fibrils seeding; **B)** Cumulative distribution of the TUJ1-positive segments length (Kruskal-Wallis test and Dunn’s multiple comparisons test: K18-Hep-4d VS Untreated p=0.0451; K18-Hep-7d VS Untreated p<0.0001; K18-NaTPP-4d VS Untreated p<0.0001; K18-NaTPP-7d VS Untreated p=0.0003).

**Supplementary Figure S9**


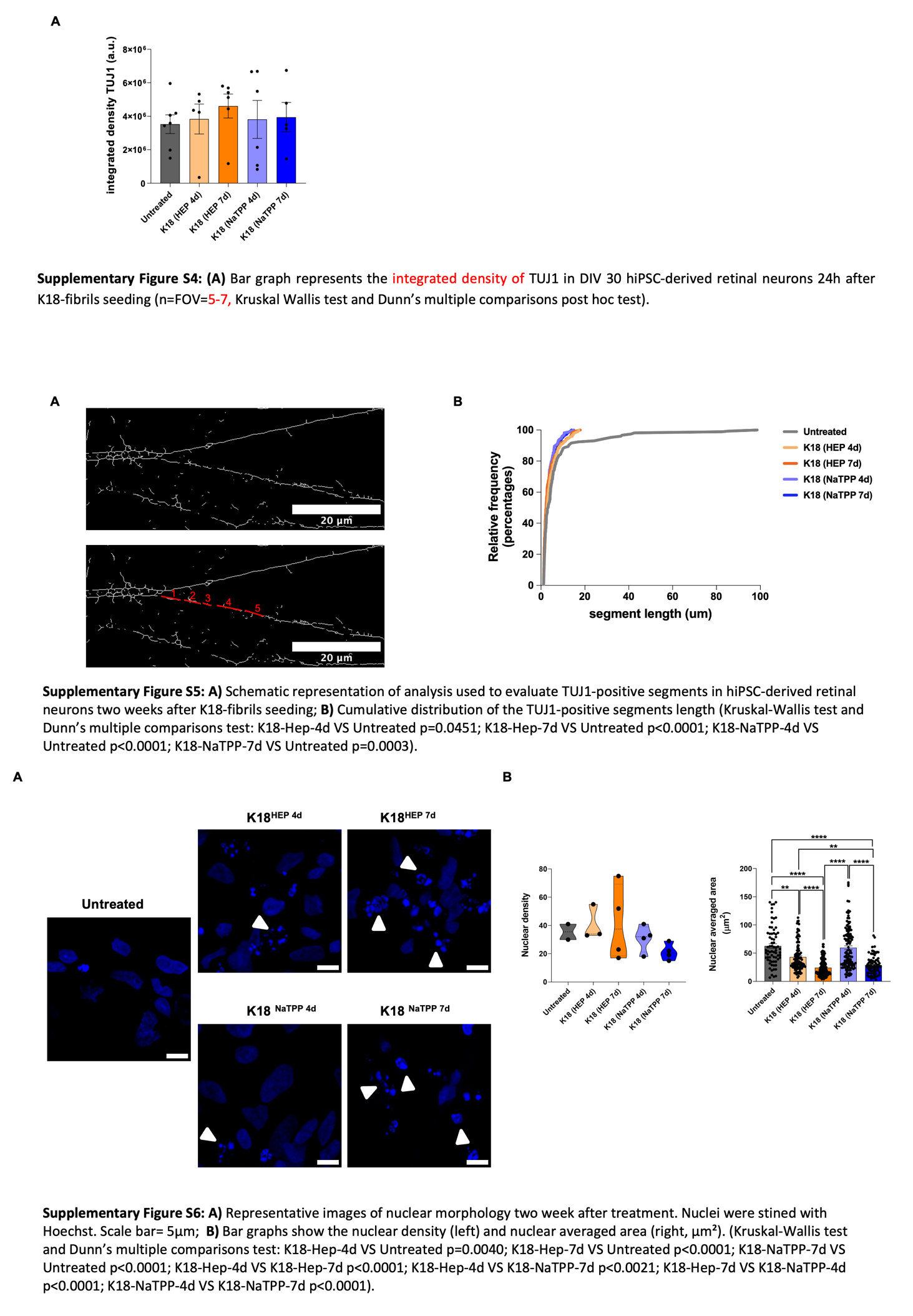


**Supplementary Figure S9:** **A)** Representative images of nuclear morphology two weeks after treatment. Nuclei were stained with Hoechst. Scale bar 5μm; B**)** Bar graphs show the nuclear density (left) and nuclear-averaged area (right, μm²). (Kruskal-Wallis test and Dunn’s multiple comparisons test: K18-Hep-4d VS Untreated p=0.0040; K18-Hep-7d VS Untreated p<0.0001; K18-NaTPP-7d VS Untreated p<0.0001; K18-Hep-4d VS K18-Hep-7d p<0.0001; K18-Hep-4d VS K18-NaTPP-7d p<0.0021; K18-Hep-7d VS K18-NaTPP-4d p<0.0001; K18-NaTPP-4d VS K18-NaTPP-7d p<0.0001).

**References**

1. Soloperto A, Quaglio D, Baiocco P, Romeo I, Mori M, Ardini M, et al. Rational design and synthesis of a novel BODIPY-based probe for selective imaging of tau tangles in human iPSC-derived cortical neurons. Sci Rep. 2022 Mar 28;12(1):5257.

2. Capocefalo A, Bizien T, Sennato S, Ghofraniha N, Bordi F, Brasili F. Responsivity of Fractal Nanoparticle Assemblies to Multiple Stimuli: Structural Insights on the Modulation of the Optical Properties. Nanomaterials. 2022 May 1;12(9):1529.

3. Salajkova Z, Barolo L, Baiocco P, Ruzicka B, Mura F, Lorenzo FD, et al. Optical signature of retinal tau fibrillation [Internet]. 2024 [cited 2024 Aug 1]. Available from: https://www.researchsquare.com/article/rs-4571431/v1

4. Barolo L, Gigante Y, Mautone L, Ghirga S, Soloperto A, Giorgi A, et al. Ferritin nanocage-enabled detection of pathological tau in living human retinal cells. Sci Rep. 2024 May 21;14(1):11533.

5. Montgomery KM, Carroll EC, Thwin AC, Quddus AY, Hodges P, Southworth DR, et al. Chemical Features of Polyanions Modulate Tau Aggregation and Conformational States. J Am Chem Soc. 2023 Feb 22;145(7):3926–36.

6. Jorgensen WL, Chandrasekhar J, Madura JD, Impey RW, Klein ML. Comparison of simple potential functions for simulating liquid water. The Journal of Chemical Physics. 1983 Jul 15;79(2):926–35.

7. Bussi G, Donadio D, Parrinello M. Canonical sampling through velocity rescaling. The Journal of Chemical Physics. 2007 Jan 7;126(1):014101.

8. Parrinello M, Rahman A. Crystal Structure and Pair Potentials: A Molecular-Dynamics Study. Phys Rev Lett. 1980 Oct 6;45(14):1196–9.

9. Hess B, Bekker H, Berendsen HJC, Fraaije JGEM. LINCS: A linear constraint solver for molecular simulations. J Comput Chem. 1997 Sep;18(12):1463–72.

10. Cheatham TEI, Miller JL, Fox T, Darden TA, Kollman PA. Molecular Dynamics Simulations on Solvated Biomolecular Systems: The Particle Mesh Ewald Method Leads to Stable Trajectories of DNA, RNA, and Proteins. J Am Chem Soc. 1995 Apr;117(14):4193–4.

11. Richards FM. AREAS, VOLUMES, PACKING, AND PROTEIN STRUCTURE. Annu Rev Biophys Bioeng. 1977 Jun;6(1):151–76.

12. Sluch VM, Chamling X, Liu MM, Berlinicke CA, Cheng J, Mitchell KL, et al. Enhanced Stem Cell Differentiation and Immunopurification of Genome Engineered Human Retinal Ganglion Cells. Stem Cells Translational Medicine. 2017 Nov 1;6(11):1972–86.

13. Ferraro G, Gigante Y, Pitea M, Mautone L, Ruocco G, Di Angelantonio S, et al. A model eye for fluorescent characterization of retinal cultures and tissues. Sci Rep. 2023 Jul 6;13(1):10983.

14. Heo CE, Han JY, Lim S, Lee J, Im D, Lee MJ, et al. ATP Kinetically Modulates Pathogenic Tau Fibrillations. ACS Chem Neurosci. 2020 Oct 7;11(19):3144–52.

15. Piacentini R, Li Puma DD, Mainardi M, Lazzarino G, Tavazzi B, Arancio O, et al. Reduced gliotransmitter release from astrocytes mediates tau‐induced synaptic dysfunction in cultured hippocampal neurons. Glia. 2017 Aug;65(8):1302–16.

16. Usenovic M, Niroomand S, Drolet RE, Yao L, Gaspar RC, Hatcher NG, et al. Internalized Tau Oligomers Cause Neurodegeneration by Inducing Accumulation of Pathogenic Tau in Human Neurons Derived from Induced Pluripotent Stem Cells. J Neurosci. 2015 Oct 21;35(42):14234–50.

17. Karikari TK, Nagel DA, Grainger A, Clarke-Bland C, Hill EJ, Moffat KG. Preparation of stable tau oligomers for cellular and biochemical studies. Analytical Biochemistry. 2019 Feb;566:67–74.
